# Monitoring Protein Secretion in *Streptomyces* Using Fluorescent Proteins

**DOI:** 10.3389/fmicb.2018.03019

**Published:** 2018-12-07

**Authors:** Mohamed Belal Hamed, Kristof Vrancken, Bohdan Bilyk, Joachim Koepff, Renata Novakova, Lieve van Mellaert, Marco Oldiges, Andriy Luzhetskyy, Jan Kormanec, Jozef Anné, Spyridoula Karamanou, Anastassios Economou

**Affiliations:** ^1^Department of Microbiology and Immunology, Rega Institute, KU Leuven, Leuven, Belgium; ^2^Molecular Biology Department, National Research Centre, Dokki, Egypt; ^3^PharmBioTec GmbH, Saarbrücken, Germany; ^4^IBG-1: Biotechnology, Institute of Bio- and Geosciences, Forschungszentrum Jülich GmbH, Jülich, Germany; ^5^Institute of Molecular Biology, Slovak Academy of Sciences, Bratislava, Slovakia; ^6^Helmholtz-Zentrum für Infektionsforschung GmbH, Braunschweig, Germany

**Keywords:** eGFP, mRFP, protein secretion, signal peptide, *Streptomyces lividans*, protein secretion biotechnology

## Abstract

Fluorescent proteins are a major cell biology tool to analyze protein sub-cellular topology. Here we have applied this technology to study protein secretion in the Gram-positive bacterium *Streptomyces lividans* TK24, a widely used host for heterologous protein secretion biotechnology. Green and monomeric red fluorescent proteins were fused behind Sec (SP^Sec^) or Tat (SP^Tat^) signal peptides to direct them through the respective export pathway. Significant secretion of fluorescent eGFP and mRFP was observed exclusively through the Tat and Sec pathways, respectively. Plasmid over-expression was compared to a chromosomally integrated *sp^Sec^-mRFP* gene to allow monitoring secretion under high and low level synthesis in various media. Fluorimetric detection of SP^Sec^-mRFP recorded folded states, while immuno-staining detected even non-folded topological intermediates. Secretion of SP^Sec^-mRFP is unexpectedly complex, is regulated independently of cell growth phase and is influenced by the growth regime. At low level synthesis, highly efficient secretion occurs until it is turned off and secretory preforms accumulate. At high level synthesis, the secretory pathway overflows and proteins are driven to folding and subsequent degradation. High-level synthesis of heterologous secretory proteins, whether secretion competent or not, has a drastic effect on the endogenous secretome, depending on their secretion efficiency. These findings lay the foundations of dissecting how protein targeting and secretion are regulated by the interplay between the metabolome, secretion factors and stress responses in the *S. lividans* model.

## Introduction

Protein export is a vital process in all cells including bacteria (Tsirigotaki et al., 2017). In the absence of outer membranes, the Gram-positive bacteria Streptomycetes, secrete proteins directly into the medium. *Streptomyces lividans*, a useful Gram-positive secretion model, can secrete several heterologous polypeptides of bacterial and eukaryotic origin (Pozidis et al., 2001; Hong et al., 2003; Lara et al., 2004; Ogino et al., 2004; Sianidis et al., 2006; Hamed et al., 2017; Kashiwagi et al., 2017). The absence of lipopolysaccharides, the availability of advanced genetic tools (Kieser et al., 2000; Kashiwagi et al., 2017), low protease activity, established industrial bioprocessing as a major producer of antibiotics (Ndlovu et al., 2015), synthetic biology tools (Phelan et al., 2017) and the avoidance of inclusion body formation, renders *S. lividans* secretion an attractive biotechnology platform. In many instances, it can provide alternative solutions when established workhorses, like *Escherichia coli*, fail (Anné et al., 2017).

Commonly, heterologous genes are fused to transcription elements and signal peptide sequences from highly expressed/secreted endogenous *Streptomyces* proteins (Lammertyn and Anné, 1998; Lammertyn et al., 1998; Anné et al., 2017), such as the subtilisin inhibitor gene (*vsi*) of *Streptomyces venezuelae* CBS762.70 (Van Mellaert et al., 1998a). The resulting proteins are thus targeted to the Sec pathway and very efficiently secreted. Examples include: active trimeric murine tumor necrosis factor alpha (Lammertyn et al., 1997; Pozidis et al., 2001), a *Jonesia* sp. xyloglucanase of 100 kDa (Sianidis et al., 2006), an extremely thermostable cellulase (Hamed et al., 2017), phospholipase D (Ogino et al., 2004), transglutaminase, β-1,4-endoglucanase and β-glucosidase (Noda et al., 2010). In similar approaches, targeting to the Tat pathway has been achieved but generally with lower yields (Schaerlaekens et al., 2004a; Gauthier et al., 2005; Anné et al., 2012; Gullon et al., 2015a).

One consistent observation in heterologous protein secretion studies as well as in proteomics of the secretome, was that protein secretion in *Streptomyces* appears to be remarkably dynamic (Hamed et al., 2017; Busche et al., 2018; Tsolis et al., unpublished). This suggested complex regulation to an extent unknown in the bacterial secretion model, *E. coli*. Moreover, protein secretion appears to be inversely correlated with directing carbon flow to biomass production. In certain media that promote poor cell growth, avid secretion of both indigenous and heterologous proteins is observed (Hamed et al., 2017; Tsolis et al., unpublished).

To dissect this complex regulatory phenotype, reporter enzymes can be used. In Gram-negative bacteria two commonly used proteins whose activity requires an extracytoplasmic location are the alkaline phosphatase PhoA, and the TEM β-lactamase BlaM (Broome-Smith et al., 1990; Gibson and Caparon, 2002; Choi and Lee, 2004; McCann et al., 2007; Belin, 2010; Saio et al., 2014). However, these reporters are unsuitable for Gram-positives as both display little activity and/or are less stable, probably because of improper folding (Broome-Smith et al., 1990; Pearce et al., 1993; Ishihara et al., 1995). PhoA and BlaM folding requires disulfide bond formation that is catalyzed by periplasmic Dsb proteins (Broome-Smith et al., 1990; Bardwell et al., 1991; Kamitani et al., 1992), that are commonly absent from Gram-positives (Reardon-Robinson and Ton-That, 2015).

In actinobacteria, the *Staphylococcus aureus* secreted nuclease (Nuc) has also been used as a reporter (Poquet et al., 1998; Downing et al., 1999). However, while Nuc activity assays are sensitive and quantitative on solid media, they cannot be used in liquid cultures. Reporters for Tat-dependent secretion in *S. lividans* include the native Tat substrates agarase (Widdick et al., 2006) and xylanase C (Faury et al., 2004). While both can be detected via simple enzymatic assays, they do not allow for in-line measurements and require off-line analysis.

Fluorescent proteins offer an alternative to enzymes. Sec-routed superfolder GFP (Aronson et al., 2011) and Tat-routed GFP (Santini et al., 2001) can be secreted in active, fluorescent conformations in *E. coli*.

To dissect the targeting and secretion regulatory mechanism in *S. lividans*, we developed a rapid, fluorescence-based assay for protein secretion that can monitor both the Sec and the Tat systems and at the same time report on the folding status of the proteins. For this, we fused enhanced GFP (eGFP) or monomeric RFP (mRFP) proteins behind Tat or Sec signal peptides. To monitor non-folded, non-fluorescent states intracellularly, we used specific antibodies. We show that eGFP and mRFP can be synthesized at high levels from pIJ486 plasmid derivatives and are excellent reporters for the Tat and Sec system, respectively, and as in *E. coli* (Tullman-Ercek et al., 2007), each making almost exclusive use of their preferred pathway. Sec-dependent mRFP secretion was among the highest observed to date for the *S. lividans* Sec system (up to 300 mg/L). mRFP secretion under low expression regimes was studied with the *sp^Sec^-mRFP* gene chromosomally integrated in single copies.

These tools allowed us to dissect the Sec pathway, both intra- and extracellularly. Sec secretion is controlled in complex ways in various media and it is not a constitutive process. Media optimal for secretion allow secretion without visible cytoplasmic intermediates. In contrast, media that lead to poor secretion, lead to (the) accumulation of secreted proteins in the cytoplasm and may cause a stress response. These data indicate the existence of complex traffic regulation that decides if a molecule will be secreted or not, while secretion overall does not cease. Therefore, it appears that this process is regulated post-transcriptionally in a way that is specific to certain molecules and not to others. Our study lays the foundations for future dissection of these sophisticated regulatory mechanisms in the *S. lividans* model.

## Results

To develop fluorescent protein reporters for secretion in *S. lividans*, the genes for the reporter proteins mRFP and eGFP were cloned in a high copy plasmid pIJ486 behind the Tat-dependent signal peptide XlnC and the Sec-dependent signal peptide Vsi (see section “Materials and Methods”) to form pIJ486-*sp^Tat^-eGFP*, pIJ486-*sp^Tat^-mRFP*, pIJ486-*sp^SecV^-eGFP*, and pIJ486-*sp^SecV^-mRFP*, respectively. The secretion of these proteins was tested in various media.

### eGFP as a Reporter Protein for Tat-Dependent Secretion in *S. lividans*

We first tested the enhanced green fluorescent protein (eGFP) as a possible reporter protein for *S. lividans* TK24 secreted in an active form, and in detectable quantities in the culture medium.

**FIGURE 1:**
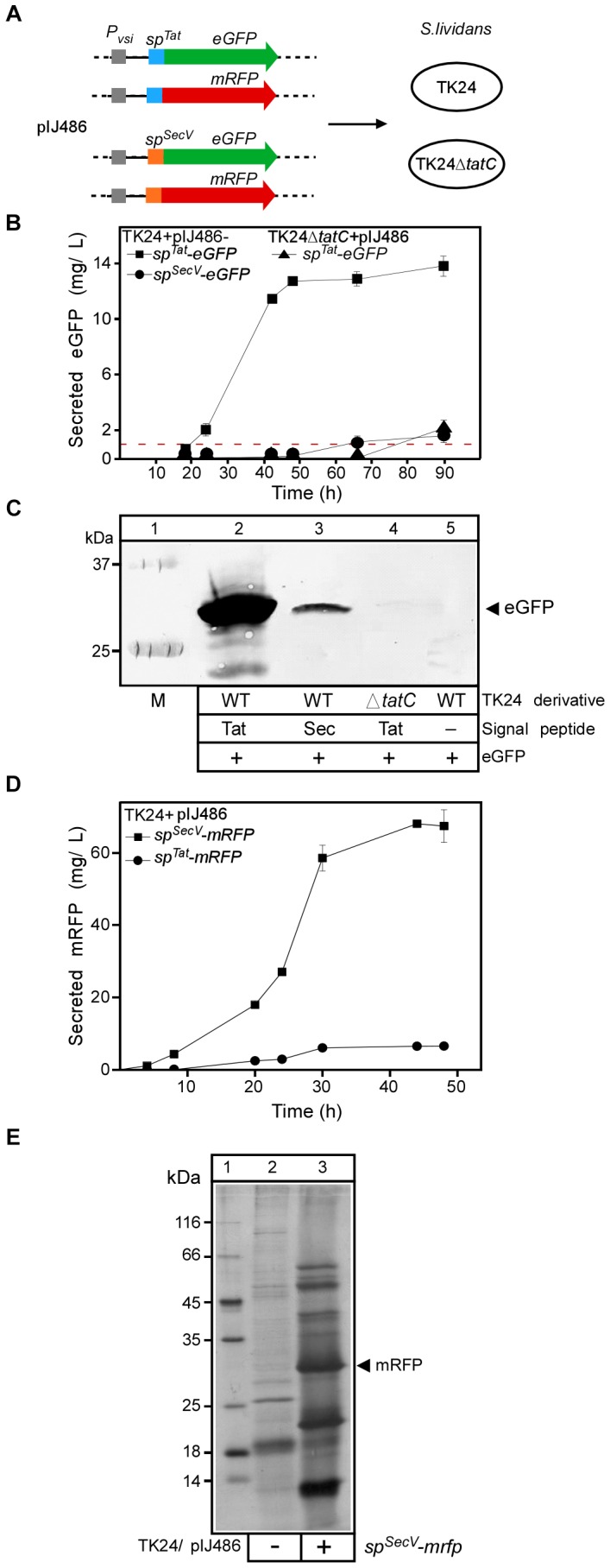
Analysis of Tat- and Sec-secreted reporter proteins for *S. lividans* TK24. **(A)** Schematic representation of the fluorescent protein constructs used in this study. **(B)** Concentration of active eGFP in the medium of the indicated strains after 18 to 90 h of growth, determined by using an eGFP calibration curve and scanning densitometry. Red line, represents the percentage of eGFP that is calculated to be derived from cell lysis (see Supplementary Materials and Methods). *n* = 3, values represent the mean ± SD. **(C)** Evaluation of eGFP accumulation in the spent culture medium (NB; 50 ml; 24 h) of the indicated strains. Proteins present in the supernatants were TCA-precipitated, separated by 12.5% SDS–PAGE and visualized by Western blotting using eGFP-antibodies. Lane 1: molecular weight markers. **(D)** The amount of secreted mRFP in (mg/L) produced by TK24 carrying pIJ486-*sp^SecV^-mRFP* or pIJ486-*sp^Tat^-mRFP* nutrient broth (NB) for the indicated time (4 to 48 h). *n* = 3, values represent the mean ± SD.**(E)** Silver-stained proteins following SDS–PAGE of TK24/pIJ486 and TK24/pIJ486-*sp^SecV^-mRFP*. The arrow indicates the mRFP protein. Cultures were grown for 48 h in NB medium after which 30 μl of filtered supernatant was loaded on the gel. Lane 1, molecular weight markers: β-galactosidase (116 kDa), bovine serum albumin (66.2 kDa), ovalbumin (45 kDa), lactate dehydrogenase (35 kDa), restriction endonuclease Bsp98I (25 kDa), β-lactoglobulin (18.4 kDa), lysozyme (14.4 kDa).

To test this, we cloned the enhanced GFP (eGFP) gene (Cormack et al., 1996) behind the strong, constitutive *vsi* promoter and the signal sequence of either the Tat-routed XlnC (*sp^Tat^-eGFP*) or the Sec-routed Vsi (*sp^SecV^-eGFP*) proteins (Figure 1A). The respective fusions were cloned into vector pIJ486 and the resulting plasmids were transformed into TK24 and TK24Δ*tatC*. The latter strain shows no Tat-dependent secretion (Schaerlaekens et al., 2001) and verifies that eGFP is routed to the Tat and not to the Sec pathway. Next, all strains were grown in NB medium and the fluorescence in the spent growth medium was measured at different time points (Figure 1B).

To quantify the amount of eGFP corresponding to a certain fluorescence readout, a calibration curve was made by measuring the fluorescence intensity of known amounts of purified recombinant eGFP (Supplementary Figure S1B) (see section “Materials and Methods”). Then the fluorescence measurements were compared with the amount detected via immuno- blotting. Fluorescence could be measured in the spent medium of cultures of TK24 cells synthesizing SP^Tat^-eGFP after 24 h of growth (38.5 relative fluorescence units, RFU, corresponding to 2.5 mg of eGFP/L) and peaked after 48 h (13 mg/L; Figure 1B), suggestive of proficient secretion of eGFP compared to a previous effort (Vrancken et al., 2007). Secretion was also better than that for human proteins tumor necrosis factor (TNF) α and interleukin (IL) (1.6 and 4.8 μg/L, respectively) when expressed behind the same promoter and secreted by the same signal peptide (Schaerlaekens et al., 2004a). Culture filtrates were sampled after 24 h, analyzed by SDS–PAGE followed by Western blotting and α-eGFP immuno-staining (Figure 1C). Significant amounts of a protein with a molecular mass of ∼30 kDa, close to the theoretical one expected for eGFP (∼27 kDa), were immuno-stained in the extracellular fraction of TK24/pIJ486-*sp^Tat^-eGFP* (lane 2).

Almost no fluorescence was measured in the spent media of strains TK24Δ*tatC/*pIJ486-*sp^Tat^-eGFP* or TK24/pIJ486-*sp^SecV^-eGFP* until 50 h of growth. The extracellular fractions of TK24/pIJ486*-sp^SecV^-eGFP* yielded very low levels of immuno-stained eGFP (lane 3), while those of TK24Δ*tatC/pIJ486-sp^Tat^-eGFP* yielded no detectable immuno-stained signal. A very low level of fluorescent signal was present at the later time points of both cultures (∼35 and 40 RFU at 90 h, corresponding to 1.7 and 2.1 mg eGFP/L, respectively), but given the delay in appearance it might be due to lysis of the mycelium (Manteca et al., 2006). To systematically quantify with high accuracy the background of polypeptides derived from potential “cell lysis” in our study, we developed a strain that synthesizes the fluorescent protein mCherry in its cytoplasm without being able to secrete it, due to the absence of a signal peptide (Supplementary Figure S1C). Detailed measurements across multiple sampling points of the growth phase were carried out in all media tested in this study and ranged from 5–10% of the total cytoplasmic content of mCherry being lost to the medium at 48 h of growth (Supplementary Figure S1D and Figure 1B, red line). These values which represent the amounts that derive from cell lysis are significantly lower than the amounts of eGFP found in the spent growth media of the two strains and would indicate that a low level of secretion is indeed taking place at the late time points. As the secreted eGFP polypeptide amounts determined by immunostaining in the spent growth medium were in proportion to those derived from their fluorescence emission, we concluded that the eGFP proteins that are secreted, are also properly folded.

Collectively, these data showed that eGFP is secreted efficiently by the Tat system, in significant amounts and that its quantification is rapid and simple. These properties make it a good reporter protein for Tat-dependent protein secretion in *S. lividans* as has been shown for *E. coli* (Thomas et al., 2001). In contrast, and in agreement with pathway specificities in *E. coli* (Lee et al., 2006), eGFP using a Tat signal peptide, does not use the Sec pathway, as revealed by the Δ*tatC* mutant. Finally, secretion through the Sec pathway of eGFP that harbors a Sec signal peptide is very inefficient (see below), when compared to that through the Tat pathway.

### mRFP as a Reporter Protein for Sec-Dependent Secretion in *S. lividans*

We next sought to develop a similar model protein to study Sec-dependent protein secretion in *S. lividans*. Several fluorescent proteins are available, in many different colors, derived from the original *Aequorea victoria* jellyfish GFP or from closely related species (Stepanenko et al., 2008). Monomeric red fluorescent protein (mRFP), obtained through *in lab* evolution of the tetrameric DsRed protein from the reef coral *Discosoma* (Campbell et al., 2002; Shaner et al., 2004), is secreted in an actively fluorescent form to the periplasm of *E. coli* when fused to YaeL, DsbA, or Maltose Binding Protein (Chen et al., 2005). Whether mRFP could also fold into an active conformation, in sufficient quantities and remain stable in the extracellular medium of *S. lividans* was tested directly.

For this, we fused the *vsi*-encoded signal sequence to the ORF of the *mRFP* gene (*sp^SecV^-mRFP*) and cloned it behind the *vsi* promoter in pIJ486 (Figure 1A). To investigate whether secretion of mRFP was strictly Sec-dependent, a fusion was also made to the signal sequence of the Tat-routed *xlnC* (*sp^Tat^-mRFP*). Both fusion-encoding genes were ligated into vector pIJ486 and the resulting constructs were transformed into TK24 and TK24Δ*tatC* cells. Next, all strains were grown in 24-well plates, with continuous shaking and fluorescence measurement in NB medium (Figure 1D).

SP^SecV^-mRFP is secreted through the Sec pathway and becomes folded, as evidenced by the fluorescence of its mRFP mature domain. SP^Tat^-mRFP is also secreted through the Tat pathway, but ∼eightfold less efficiently than SP^SecV^-mRFP through the Sec pathway (∼8 vs. ∼65 mg/L, respectively) (Figure 1D). The observed low-level secretion of SP^Tat^-mRFP was Tat-dependent as TK24Δ*tatC* could not secrete it at all (∼0.13 mg/L). This also demonstrated that there is no detectable cross-talk between the Tat and Sec pathways for this fusion protein. These observations also corroborate previous findings that secretion yields from the TK24 Sec pathway are superior to those from Tat, for most proteins (Schaerlaekens et al., 2004b; Gullon et al., 2015a).

### Quantification of mRFP Secreted by *S. lividans*

**FIGURE 2:**
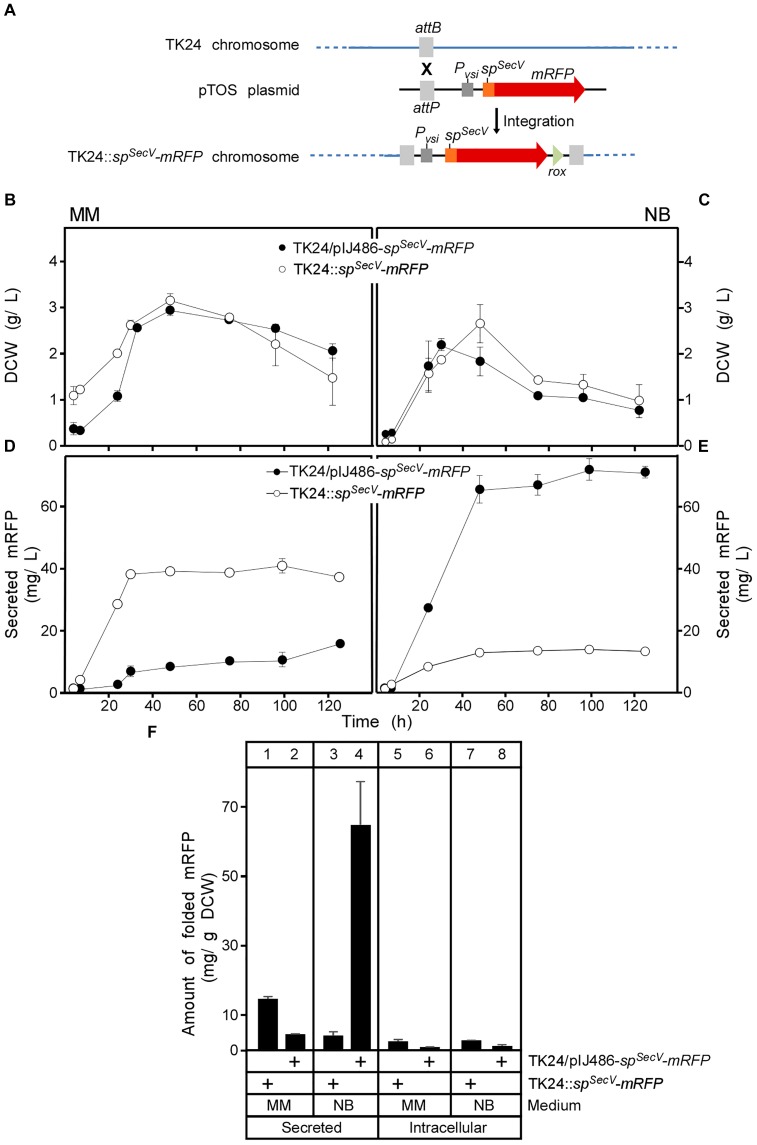
Integration of the *sp^SecV^-mRFP* gene into the chromosome of *S. lividans* TK24. **(A)** The VWB integrase catalyzes integration of the pTOS+*sp^SecV^-mRFP* into the VWB-attachment site of TK24. **(B**,**C)** Comparison of cell growth of TK24 with integrated *sp^SecV^-mRFP* or carrying the plasmid-borne copy of *sp^SecV^-mRFP* in minimal medium (MM) and nutrient broth (NB) expressed as values of dry cell weight (DCW) (g/L). *n* = 3, values represent the mean ± SD. **(D**,**E)** Amounts of mRFP secreted (in mg/L) from TK24 with integrated *sp^SecV^-mRFP* or carrying the plasmid-borne copy of *sp^SecV^-mRFP* in minimal medium (MM) and nutrient broth (NB) for the indicated time related to its growth curves in the same media. *n* = 3, values represent the mean ± SD. **(F)** Comparison of the yield of secreted and intracytoplasmic folded mRFP determined in (mg) correlated to a gram of DCW at 48 h from *S. lividans* TK24 with integrated *sp^SecV^-mRFP* or carrying the plasmid-borne copy of *sp^SecV^-mRFP* in minimal medium (MM) and nutrient broth (NB). *n* = 3, values represent the mean ± SD.

SP^SecV^-mRFP secretion could clearly be detected in the culture supernatant of cells growing in NB medium (Figure 1E) which turned red after a couple of days (Supplementary Figure S6G). To quantify the secreted mRFP, we cloned *mRFP* as a His-tagged version in pIMBB643, transformed it in *E. coli* strain BL21 and purified His-mRFP by metal affinity chromatography (not shown). We then generated calibration curves of both fluorescence measurements and protein mass as for eGFP (Supplementary Figure S1A). Finally, the amounts detected by fluorescence measurements with those detected by western immuno-staining were in close agreement. These experiments revealed that secreted mRFP amounts in spent NB media of ∼100–300 mg/L after 48 h of growth, depending on the NB medium batch (See Figure 2E and Supplementary Figure S6C). These are among the highest levels of Sec secretion of heterologous proteins known for *S. lividans* (Vrancken and Anné, 2009; Kashiwagi et al., 2017; Hamed et al., 2018).

### Chromosomal Integration of the mRFP Reporter Gene

For enhanced stability in some applications, including long fermentation regimes and to avoid constant use of antibiotic selection, heterologous genes can be integrated into the *Streptomyces* chromosome (Hong and Hondalus, 2008). This also allows the study of mRFP secretion at a stable low level synthesis regime. To prepare such a strain we integrated *sp^SecV^-mRFP* in the chromosome of TK24 using the phage VWB attachment site (Van Mellaert et al., 1998b) to form TK24::*sp^SecV^-mRFP* (Figure 2A and Supplementary Figure S2). The derivative strain (TK24::*sp^SecV^-mRFP*) grew as well as wild-type TK24 in 250 mL flasks (Figures 2B,C and Supplementary Figures S5A,B) suggesting similar fitness. Strong mRFP fluorescence signals equivalent to secretion of 38 mg/L were measured after 48 h in supernatants of this strain in Minimal Medium (Figures 2D,F, lane 1), demonstrating efficient secretion when the gene is chromosomally integrated, even better than that of TK24/pIJ486-*sp^SecV^-mRFP* (Figures 2D,F, lane 2). In contrast, secretion in NB medium, generally one of the best secreting media for TK24 in our experience (Hamed et al., 2017), was eight times lower in TK24::*sp^SecV^-mRFP* than in TK24/*sp^SecV^-mRFP* (Figures 2E,F, compare lane 4 to 3). Analysis of the spent growth medium of TK24::*sp^SecV^-mRFP* indicated a prominent band of ∼27 kDa, close to the theoretical mass of mRFP (25.4 kDa) and which cross-reacted with the mRFP antibody (see fluorescence assay section in “Materials and Methods”). Similar results were obtained when *mRFP* was integrated behind the native promoter and signal peptide of the endogenous *lsi* gene (*S. lividans* subtilisin inhibitor) *sp^SecL^-mRFP* (Supplementary Figures S3, S4, S5C,D).

To better understand the mechanisms that regulate secretion for a given protein in different media, we probed the cytoplasmic contents of the two derivative strains TK24/pIJ486-*sp^SecV^-mRFP* and TK24::*sp^SecV^-mRFP*. This analysis revealed that significant amounts of cytoplasmic forms of the secretory protein were observed irrespective of final secretion yields. We dissect this in depth below.

### Testing of Different Growth Media to Study mRFP Secretion

Growth media have a major effect on protein secretion in *S. lividans*, with secretion being inversely correlated to biomass increase and the secretome showing dynamic changes (Hamed et al., 2017; Busche et al., 2018; Tsolis et al., unpublished). The availability of various media provided us with a means to dissect mechanisms relating to regulation of protein secretion and monitoring protein flow from the cytoplasm to the secretory pathway. We examined mRFP secretion in five different growth media: Tryptic soy broth (TSB), Bennet medium (Ben), Minimal medium (MM), Minimal medium supplemented with casamino acids (5 g/L; MMC), nutrient broth (NB), and complete medium (CM) supplemented with glucose, glycerol, or xylose (16 g/L).

Cell growth in the presence or absence of *sp^SecV^-mRFP* was similar, although in some media cells expressing the heterologous protein generated less biomass (Supplementary Figures S6A,B). Based on the final yields of secreted mRFP per liter and dry cell weight determinations, we could normalize the secretion per gram of dry cell weight and therefore compare it across growth conditions. All media could be classified in three categories: those generating (a) low (MM and MMC), (b) moderate (Bennet and TSB) or (c) high (NB and CM/glucose) secreted mRFP yields (Table 1 and Supplementary Figures S6D,E). Based on this analysis we focused hereafter on MM and NB as representative media that promote two distinct extreme secretion states and analyzed TK24::*sp^SecV^-mRFP* (hereafter: low SP^Sec^-mRFP levels synthesized) and TK24/pIJ486-*sp^SecV^-mRFP* (hereafter: high SP^Sec^-mRFP levels synthesized).

**Table 1:** mRFP production and yield in different fermentation media over different phases (Late of exponential growth phase vs. late stationary phase).

	End of exponential phase		Late stationary phase	
Medium	Secreted mRFP concentration (mg/L)	Yield of secreted mRFP per unit biomass (mg/g DCW)	Secreted mRFP concentration (mg/L)	Yield of secreted mRFP per unit biomass (mg/g DCW)
MM	9.02 (±2.13)	3.9 (±0.64)	8.5 (±3.17)	3.62 (±1.44)
MMC	16.08 (±3.21)	6.03 (±1.03)	60.9 (±3.62)	20.5 (±2.46)
TSB	22.9 (±2.36)	8.6 (±3.16)	82.7 (±1.18)	44.6 (±4.57)
Bennet	7.5 (±0.45)	5.6 (±0.39)	8.02 (±1.6)	4.04 (±1.06)
NB	53.2 (±1.14)	50.7 (±8.1)	72.7 (±1.47)	64.3 (±1.86)
CM (glucose)	59.7 (±3.34)	13.6 (±0.13)	224.9 (±28.3)	52.6 (±2.23)
CM (xylose)	135.5 (±21.3)	21.8 (±3.28)	162.1 (±18.67)	25.1 (±3.32)
CM (glycerol)	106.04 ( ± 33.71)	26.4 (±7.75)	215.9 (±23.2)	34.6 (±7.89)

*MM, minimal medium; MMC, minimal medium plus casamino acids (5 g/L); TSB, tryptic soy broth; NB, nutrient broth; CM, complete medium with different carbon sources (16 g/L); DCW, dry cell weight. n = 3.*

### Secretion Efficiency of SP^SecV^-mRFP

**FIGURE 3:**
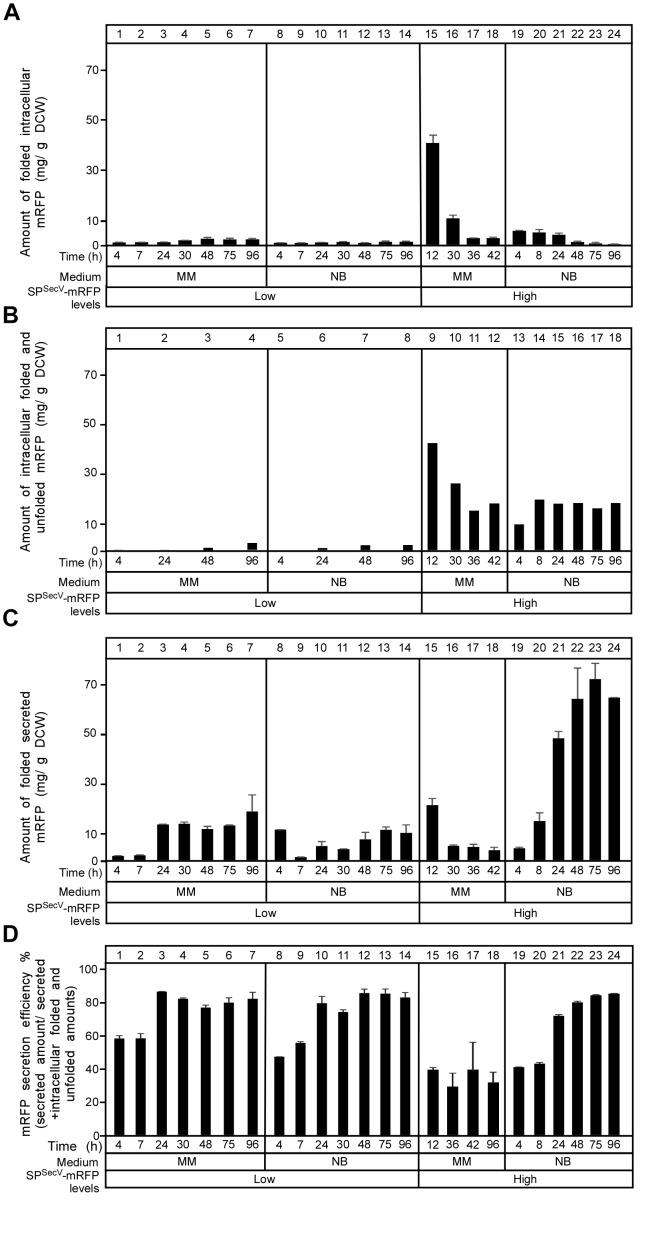
Secretion efficiency of mRFP. **(A)** The amount of intracytoplasmic folded mRFP determined in (mg) correlated to a gram of dry cell mass produced in TK24::*sp^SecV^-mRFP* (Low production level) or carrying the plasmid-borne copy of *sp^SecV^-mRFP* (High production level) in minimal medium (MM) and nutrient broth (NB) for the indicated time related to its growth curves in the same media. *n* = 3, values represent the mean ± SD. **(B)** The amount of total intracytoplasmic mRFP (folded and unfolded) in (mg) correlated to a gram of dry cell mass produced by TK24::*sp^SecV^-mRFP* (Low production level) or carrying pIJ486-*sp^SecV^-mRFP* (High production level) in minimal medium (MM) and nutrient broth (NB) for the indicated time related to its growth curves in the same media. **(C)** The amount of mRFP secreted (in mg) correlated to a gram of DCW produced by TK24::*sp^SecV^-mRFP* (Low production level) or carrying pIJ486-*sp^SecV^-mRFP* (High production level) in minimal medium (MM) and nutrient broth (NB) for the indicated time related to its growth curves in the same media. *n* = 3, values represent the mean ± SD. **(D)** Secretion efficiency, expressed as (%) of the total detectable secreted and folded mRFP as a fraction of the total synthesized SP^SecV^-mRFP (folded secreted+folded cytoplasmic+non-folded cytoplasmic), of TK24::*sp^SecV^-mRFP* (Low production level) or carrying pIJ486-*sp^SecV^-mRFP* (High production level) in minimal medium (MM) and nutrient broth (NB) for the indicated time related to its growth curves in the same media. *n* = 3, values represent the mean ± SD.

To monitor the flow of SP^SecV^-mRFP along the secretion pathway and to identify possible cytoplasmic intermediates, we determined the total amount of SP^SecV^*-*mRFP synthesized, and determined its distribution in folded cytoplasmic (Figure 3A; determined by mRFP fluorescence), total cytoplasmic (Figure 3B; folded and non-folded protein; determined by immuno-staining) and extracellularly folded (Figure 3C) forms.

After determining the total amounts of intracytoplasmic mRFP by immuno-staining for mRFP on western blots (in Figure 3B; see below), we could subtract the amounts of folded mRFP (derived from Figure 3A). This revealed that cells synthesizing high levels of SP^SecV^-mRFP in either MM (Figure 3B, lanes 9–12) or NB (lanes 13–18), also retain high levels of intracellular protein that is not properly folded. We performed a similar analysis on secretome polypeptides. However, in these extracts we always found that the mRFP amounts that were determined by immuno-staining were similar to those determined by fluorescence detection. Therefore, we concluded that most of the detectable secreted mRFP was also properly folded.

This analysis revealed that:

(a) in MM the overall SP^SecV^-mRFP synthesized in the “low level” expressing strain is twice that in the “high level” plasmid-bearing strain. Within 24 h of growth >80% of the low, but only ∼50% of the high level, SP^SecV^-mRFP synthesized in MM is being secreted. In MM medium, mRFP secretion levels off at 24 h, while exponential cell growth continues (see Figure 2B).
(b) In the high level SP^SecV^-mRFP synthesized in MM, secretion of more than 50% is not observed, indicating that the export capacity of the cell is saturated or other mechanisms prevent it.
(c) the SP^SecV^-mRFP synthesized either at low or high level overall in NB medium is secreted to ∼80%, gradually over 48 h and then levels off.

Collectively, these data define the “efficiency of secretion” as the total detectable secreted and folded mRFP as a fraction of the total SP^SecV^-mRFP synthesized (folded secreted+folded cytoplasmic+non-folded cytoplasmic) (Figure 3D).

We concluded that at low levels of synthesis in MM, most SP^SecV^-mRFP is directed for secretion, while at high levels, the secretion system is saturated and almost half the protein is directed for cytoplasmic folding. In the highest performing NB medium, most of the high level SP^SecV^-mRFP is directed to secretion, but significant amounts are still detected in the cytoplasm. As these would be detected by immuno-staining and do not yield equivalent amounts of fluorescence, most of these polypeptides would be non-folded. This indicated that it is not the synthesized amounts *per se* that lead to suboptimal secretion but rather the medium context in which this happens. This implied a particular cellular response to some of the nutrients in NB and that these would facilitate targeting and/or secretion. Such nutrients, may become limiting in MM.

### Intracytoplasmic States of SP^SecV^-mRFP

**FIGURE 4:**
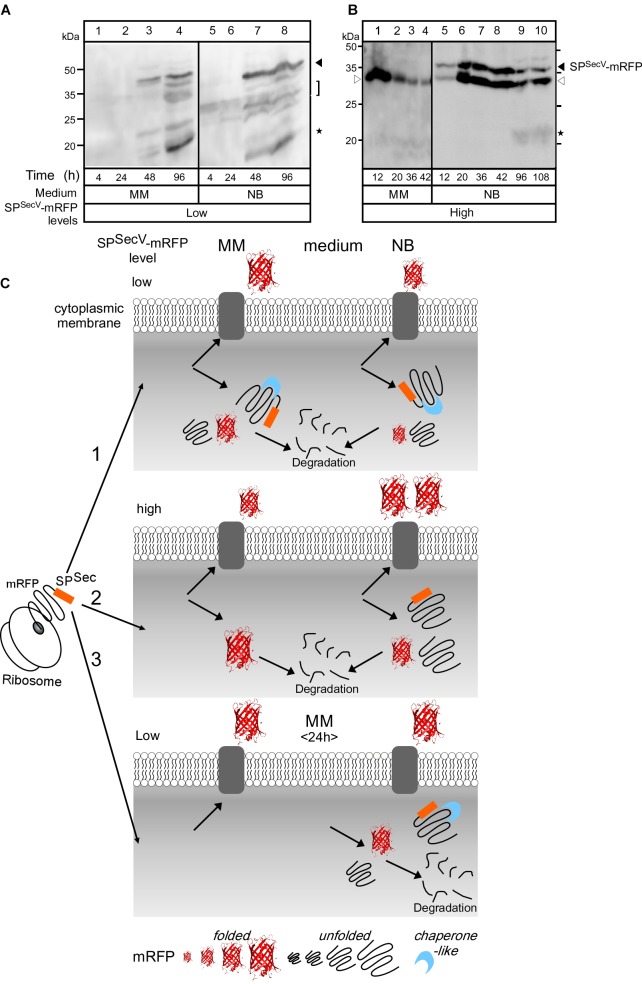
Intracellular states of mRFP **(A)** and **(B)** Western blot analysis for intracellular mRFP in TK24 grown in minimal medium (MM) and nutrient broth (NB). Total cell lysates loaded are equivalent to 0.6 mg of dry cell mass of TK24::sp^SecV^-*mRFP* (Low production level) or carrying pIJ486-sp^SecV^-*mRFP* (High production level) grown for the indicated times in the indicated media, washed twice and loaded on 12% SDS-PAGE and visualized using mRFP-antibodies. Filled arrow: intact mRFP; empty arrow: mature mRFP; asterisks: degradation of mRFP. Molecular weight markers indicted for both panels for MM (left) and NB (right): ovalbumin (50 kDa), carbonic anhydrase (34 kDa), -lactoglobulin (26 kDa), lysozyme (20 kDa). **(C)** Schematic diagram describes the hypothesis explaining mRFP secretion. (1) TK24::sp^SecV^-*mRFP* (low level). In MM, most of the synthesized mRFP is secreted. The non-secreted mRFP is accumulated intracellularly in a stable premature form with its signal peptide and in a premature form that loses its signal peptide by proteolytic attack and forms a folded mRFP or is completely degraded. In NB, little amount of synthesized mRFP is secreted while the rest is accumulated intracellularly and shows the same behavior as in MM. (2) TK24 carrying pIJ486-sp^SecV^-*mRFP* (High production level). In MM, 50% of the synthesized mRFP is secreted and the rest is folded intracellularly and then proteolytically degraded. In NB, 90% of the synthesized mRFP is secreted and the rest is accumulated intracellularly in an unstable premature form that loses its signal peptide to convert to a folded mRFP, or becomes completely proteolytically degraded. (3) Secretion of SP^SecV^-mRFP (low level) stops at 24 h. Before 24 h, all the formed mRFP is secreted, while after 24 h mRFP begun to accumulate in the cytoplasm with the fate described above.

To further probe the cellular mechanisms that regulate protein targeting and secretion in *S. lividans* and to identify what are the non-folded intracelullar states we analyzed the intracellular species of SP^SecV^-mRFP that were detected by α-mRFP antibodies (Figures 3B, 4A,B). Antibodies allowed monitoring of even the non-folded (i.e., non-fluorescent) cytoplasmic states of SP^SecV^-mRFP (Figure 3B).

At low level synthesis in MM, there are no detectable amounts of SP^SecV^-mRFP in cytosolic extracts for up to 24 h of growth (Figure 4A, lanes 1 and 2). This agrees with the finding that at 24 h most of the protein is secreted (Figures 3C,D, lane 3; Figure 2D). Interestingly, after secretion ceased, SP^SecV^-mRFP begun accumulating in the cytoplasm at late time points of cell growth (Figure 4A, lanes 3 and 4). The protein was found in a rather stabilized preform state with its signal peptide attached (Figure 4A, lanes 3 and 4, filled arrow), but begun undergoing visible degradation by losing its signal peptide to acquire the folded mature form (bracket) or even underwent further degradation to lower mass forms (asterisks). Similar results were obtained with the SP^SecL^-mRFP derivative although in this case the preform seems to be more resistant to proteolytic attack (Supplementary Figure S5E, lanes 3 and 6). Given that SP^SecL^-mRFP is synthesized at significantly lower amounts than SP^SecV^-mRFP (compare Supplementary Figure S5F, lanes 2 and 8 to Figure 2F, lanes 5 and 7), we concluded that while in MM secretion ceases at ∼24 h of growth, the synthesis of SP^SecV^-mRFP and SP^SecL^-mRFP continues unabated. We hypothesize that althought secretion of SP^SecL^-mRFP stops but an unknown cellular factor(s) with a presumed chaperone-like activity protects these molecules through formation of stoichiometric complexes (Figure 4C).

At high level synthesis in MM, SP^SecV^-mRFP is detected in the cytoplasm in large amounts as a single polypeptide with the mass of mature mRFP (Figure 4B, lanes 1–4) that also yields a strong fluorescence signal (Figure 3A, lanes 15–18), indicative of proper folding. These intracytoplasmic amounts are particularly elevated at the earliest time-points of growth (Figure 3A, lane 15; Figure 4B, lane 1). However, this species is not stable overtime and gets degraded by >76% by 42 h (Figure 4B, lane 4).

At low level synthesis in NB (Figure 4A, lanes 5–8), the pattern was similar to that seen in MM, while at high-level synthesis (Figure 4B, lanes 5–10), the preform SP^SecV^-mRFP was detected in the cytoplasm at very elevated amounts (filled arrowhead). A species with an apparent mass slightly lower than that of the mature mRFP also accumulated (Figure 4B, empty arrowhead). We assume this to be a proteolytic product of SP^SecV^-mRFP. Given the very low levels of fluorescence signal (Figure 3A, lanes 22–24), neither one of these intracellular forms represented properly folded mRFP. The remarkable stability of the preform is suggestive of a chaperone-like factor to stabilize it, as suggested for the MM above, and perhaps insufficient amounts of an unknown cellular protease to degrade it (Figures 4A,C).

### The Effect of Heterologous SP^SecV^-mRFP Synthesis/Secretion on the Endogenous Secretome

One unexpected observation from the data above was that secretion of SP^SecV^-mRFP was apparently prevented in MM medium although the cell carried on growing exponentially, and the preform accumulated in the cytoplasm (Figure 4A lanes 3 and 4; Figure 4B, lanes 1–4). This suggested that cellular factors may have specifically prevented the secretion process of SP^SecV^-mRFP, independently of the amounts of its synthesis, while the cell kept on growing. The opposite was seen during growth in NB. While cell growth reached stationary phase after ∼24 h (Figure 2C), secretion of SP^SecV^-mRFP carried on unabated until ∼48 h (Figures 2E, 3C, lanes 19–22).

**FIGURE 5:**
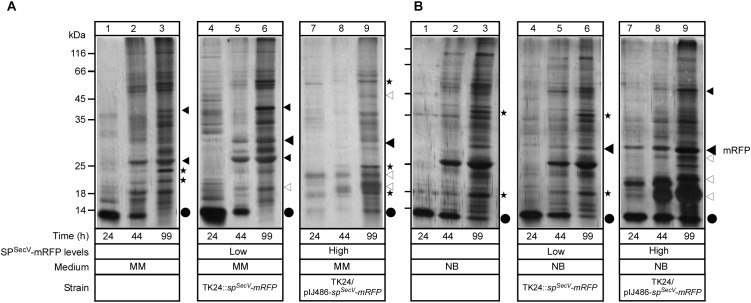
Effect of mRFP secretion on the total endogenous secretome. **(A**,**B)** Polypeptides (0.4–5 μg/lane) from culture supernatants (0.9–16 μL/lane) that are equivalent to 0.2 mg of dry cell weight from TK24, TK24::*sp^SecV^-mRFP* (Low production level) or carrying pIJ486-*sp^SecV^-mRFP* (High production level), which were grown for the indicated times in minimal medium (MM) and nutrient broth (NB), and were analyzed by SDS–PAGE and silver-staining. Lane 1, molecular weight markers as in Figure 1E. Filled big arrow, secreted mRFP; asterisks and small filled black arrows, secretome polypeptides with reduced abundance; empty arrows, new secretome polypeptides; black dots, secreted lividans subtilisin inhibitor.

These observations prompted us to monitor if secretion of the heterologous SP^SecV^-mRFP correlates with the generic export competence of the TK24 secretome. For this we harvested the secretomes of TK24 growing in MM or NB (Figures 5A,B, lanes 1–3, respectively), and compared them to those synthesizing the heterologous SP^SecV^-mRFP at low (Figures 5A,B, lanes 4–6) or high (Figures 5A,B, lanes 7–9) levels, by SDS–PAGE.

In MM, the abundance of a few TK24 proteins was reduced (Figure 5A, lanes 1–3; asterisks), while the abundance of other bands increased in the low level SP^SecV^-mRFP strain secretome (lanes 4–6; small filled arrowheads). Also, some new bands in the secretome of the low level SP^SecV^-mRFP expressing strain appeared (lanes 4–6; empty arrowheads). Nevertheless, the secretion profiles were overall very similar. In contrast, the secretome of the high level SP^SecV^-mRFP-producing strain, in the earlier time points (Figure 5A, lanes 7 and 8), showed little overall export of secretome proteins seen in TK24 (lanes 2 and 3; asterisks) and contained only a minor population of secreted mRFP. Some recovery of TK24 secretome polypeptides was seen at later time points (lane 9), together with some new exported polypeptides of high abundance (empty arrowheads).

In NB media, the secretion profile of TK24 (Figure 5B, lanes 1–3), and that of the low level SP^SecV^-mRFP-synthesizing strain (lanes 4–6) were very similar with only minor changes (asterisks). In contrast, the secretomes from high-level SP^SecV^-mRFP synthesizing cells (lanes 7–9), contained not only a strong band of secreted mRFP, but also several other unknown proteins that are secreted at high levels (empty arrowheads). Some of them are likely to be the same polypeptides as those seen in the corresponding MM secretomes (Figure 5A, lane 9).

We concluded that high-level synthesis/secretion of SP^SecV^-mRFP has a direct impact on the endogenous secretome. The molecular basis of this secretome regulation is likely to be complex and cannot be currently deconvoluted. Nevertheless, both the effects on the secretome and the intracellular proteolytic responses that were detected (Figures 4A,B), may be suggestive of a possible stress response, the severity of which is dependent on the growth medium.

### Cellular Response to Compromised Protein Secretion

**FIGURE 6:**
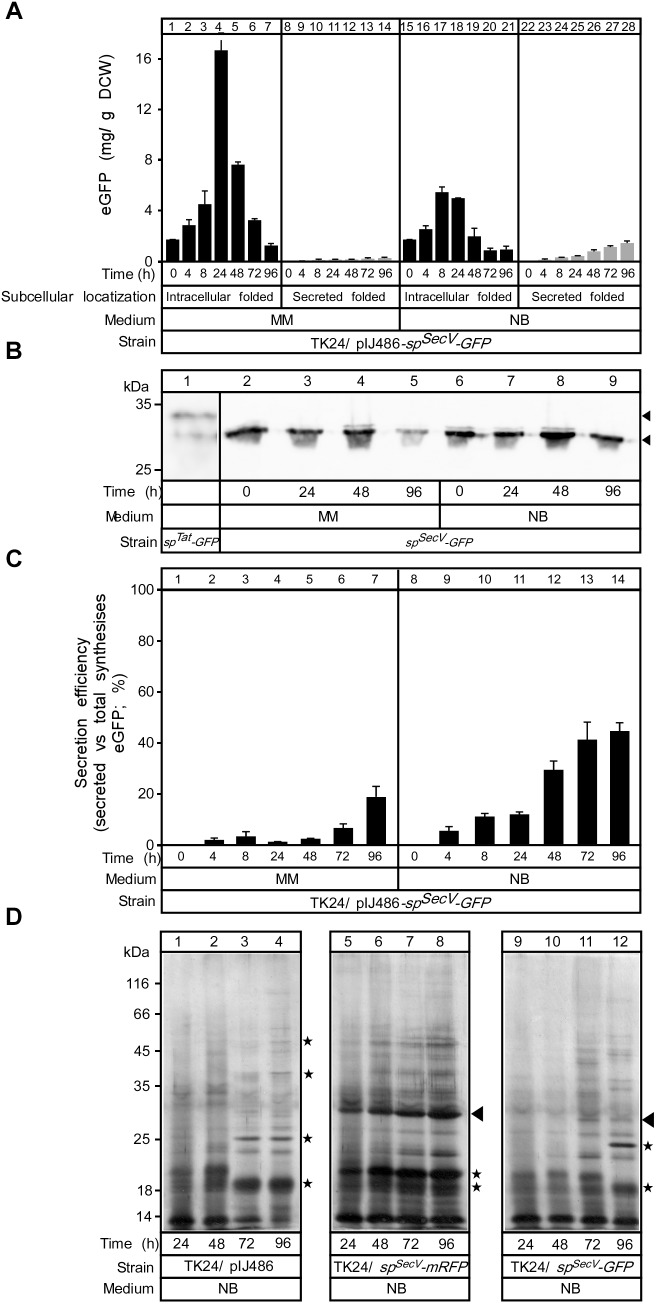
Comparing mRFP and GFP synthesis and secretion. **(A)** The amount of intracytoplasmic, folded (black bars) and secreted (gray bars) eGFP in (mg) correlated to a gram of DCW produced in TK24/pIJ486-*sp^SecV^-eGFP* in minimal medium (MM) and nutrient broth (NB) for the indicated time related to its growth curves in the same media. *n* = 3, values represent the mean ± SD. **(B)** Western blot analysis for intracellular eGFP in minimal medium (MM) and nutrient broth (NB). Total cell lysates loaded are equivalent to 0.6 mg of dry cell mass of TK24 a plasmid-borne copy of *sp^SecV^-eGFP* were grown for the indicated times in the given media. Intact and premature eGFP are indicated with filled arrow. **(C)** Secretion efficiency, expressed as (%) of the total detectable secreted and folded eGFP as a fraction of the total SP^SecV^-eGFP synthesized (folded secreted+folded cytoplasmic+non-folded cytoplasmic), of TK24/pIJ486-*sp^SecV^-eGFP* in minimal medium (MM) and nutrient broth (NB) for the indicated time (related to its growth curves in the same media). *n* = 3, values represent the mean ± SD. **(D)** Polypeptides (0.8–5 μg/lane) from culture supernatants (2–16 μL/lane) that are equivalent to 0.2 mg of dry cell mass from TK24 or TK4/pIJ486-*sp^SecV^-mRFP* or pIJ486-*sp^SecV^-eGFP* which were grown for the indicated times in nutrient broth (NB) and were analyzed by SDS–PAGE and silver-staining. Filled arrow, secreted mRFP or eGFP; asterisks, differences in secretome polypeptides.

The data above revealed a complex cellular response that results from growth in specific media and is affected by the level of the heterologous SP^SecV^-mRFP synthesis. To further probe if this response is directly related to high levels of secretion, we turned to SP^SecV^-eGFP, a protein that is synthesized at high levels but displays a very compromised secretion (Figure 1B). We monitored the fate of SP^SecV^-eGFP during growth in MM and NB media (Figure 6).

SP^SecV^-eGFP is synthesized at levels of ∼3- (MM) to ∼30-fold (NB) lower than those of SP^SecV^-mRFP (Figure 6A). Most of the synthesized eGFP accumulated inside the cell in both media, having a mass consistent with loss of the SP^SecV^ signal peptide (Figure 6B) and was fluorescent, and therefore folded. The accumulated intracellular folded GFP had the expected apparent mass of mature GFP and showed stability until 24 h of growth in both media, but after that it appeared to be proteolytically degraded (Figure 6B, lanes 5 and 9). Extremely low secreted amounts (∼0.3 and 1.5 mg/g DCW) in MM and NB media, respectively, at 48 h were identified (Figure 6A). These values led to a very low secretion efficiency for SP^SecV^-eGFP in MM (Figure 6C, lanes 1–7; ∼2% at 48 h) and a moderate one in NB (lanes 8–14; 28% at 48 h).

Analysis of the secretome profiles of TK24 synthesizing SP^SecV^-mRFP (Figure 6D, lanes 5–8) and SP^SecV^-eGFP (lanes 9–12) showed characteristic secretome changes compared to those of TK24 with an empty plasmid (lanes 1–4). The secretome of SP^SecV^-eGFP-synthesizing cells showed reduced overall amounts of total proteins compared with TK24 harboring an empty plasmid (lanes 1–4, asterisks) and became more prominent at late growth points (compare lanes 11 and 12 to lanes 3 and 4). These data suggest that although synthesis of a poorly secreted polypeptide does not affect cellular growth (Supplementary Figures S5A,B), it can influence the secretome. Given the lower levels of SP^SecV^-eGFP synthesis, these effects cannot be attributed simply to heterologous protein over-synthesis and to potential competition for secretory machineries. It might be that the fast folded eGFP blocks the secretory machineries and prevents the secretion of other proteins. The molecular basis of this response remains unknown.

## Discussion

We developed fluorescent protein reporters for monitoring Tat- and Sec-dependent protein secretion in *S. lividans*. In combination with immuno-detection, we now have a powerful system with which both folded and non-folded secreted and cytoplasmic secretory pathway intermediates can be accurately monitored.

Actively fluorescent eGFP could only be secreted after cytoplasmic folding and subsequent translocation through the Tat pathway. In contrast, Sec-dependent export was very poor (Figure 6A), as shown in *E. coli* (Lee et al., 2006). Unlike eGFP, mRFP was secreted through the Sec pathway and folded (Figure 3A). Therefore, mRFP can be used as a quantitative reporter for Sec-dependent secretion and folding in *S. lividans*. Low levels (∼10%) of SP^Sec^-mRFP were funneled to the Tat pathway (Figure 1D), as seen previously for a Sec-routed cholera toxin-mRFP in *E. coli* (Tinker et al., 2005).

Secretion differences between eGFP and mRFP are remarkable given their 33% identical/54% strongly similar amino acids and nearly identical structures (Wall et al., 2000; Shagin et al., 2004). Differences might derive from structural dynamics and folding kinetics in the cytoplasm (Stepanenko et al., 2013; Tsirigotaki et al., 2018). This could also affect fluorophore maturation patterns, shown for DsRed and eGFP (Wall et al., 2000). The poor secretion of Sec-routed eGFP suggests that only small amounts of it exist in non-folded, translocation-competent conformations necessary for Sec-routing (Tsirigotaki et al., 2017). Presumably, productive chaperone/holdase interactions that could prevent folding, are unavailable or eGFP folding kinetics are very fast and this excludes timely chaperone binding. Alternatively, eGFP gets secreted through the Sec pathway as efficiently as mRFP, but due to incorrect folding, may be more protease-susceptible. We think this is less likely given the large intracellular amounts of eGFP (Figure 6A). High levels of secretion of superfolder GFP were achieved in *E. coli* but only using co-translational targeting (Dinh and Bernhardt, 2011). These results suggest that, as in *E. coli* (Chatzi et al., 2017; Sardis et al., 2017), the ability of exported proteins to maintain non-folded states can decide Sec pathway entry. In *E. coli*, this is secured via either chaperones, like SecB, or an association with the SRP, but Gram-positive bacteria like *S. lividans*, are devoid of SecB (Ruckert et al., 2015), and the basis for such events remains unknown.

Beside their use as reporters, eGFP and mRFP provided high production yields. Tat-dependent eGFP secretion of >10 mg/L is higher than the amount of human tumor necrosis factor (hTNF) α and interleukin (IL) secreted in *S. lividans* TK24 via the Tat pathway (Vrancken et al., 2007) and Sec-dependent secretion of mRFP (∼300 mg/L or 700 mg/g DCW) is more than the Sec- dependant secretion of thermostable cellulase A (70 mg/L) (Hamed et al., 2017), xyloglucanase (100–150 mg/L) (Sianidis et al., 2006), endoglucanase from *Thermobifida fusca* (173 mg/L) (Li et al., 2013), phospholipase D (PLD) from *Streptoverticillium cinnamoneum* (118 mg/L) (Ogino et al., 2004) and two major lipoproteins (A) and (B) from *Mycobacterium tuberculosis* (80 and 200 mg/L, respectively) (Tremblay et al., 2002), while less than laccase from *S. coelicolor* A3(2) (350 mg/L) (Dube et al., 2008) and chitinase C from *S. coelicolor* A3(2) (1070 mg/L) (Nguyen-Thi and Doucet, 2016). The values obtained with mRFP make them amongst the highest yields obtained so far in *S. lividans*. Endo-xylanase from *Aspergillus nidulans* (19 U/mL) (Diaz et al., 2004) and alpha-amylase AmlB (∼70 U/mg DCW) (Gullon et al., 2015b), have also been substantially secreted, but secretion amounts have only been correlated to activities and not to secreted mass and cannot be compared at the moment. mRFP could be a useful *N*-terminal fusion partner for Sec-routed proteins that are poorly secreted (Chen et al., 2005).

Finally, both proteins offer the major advantage that they can be easily and quantitatively detected on-line during fermentation optimization or other studies. Demonstrating this potential, secretion of SP^SecV^-mRFP in CM medium, revealed a previously unknown 2-step secretion pattern (Supplementary Figure S6D lanes 13–18), probably correlated to a glucose depletion metabolic switch. SP^SecV^-mRFP can provide a means for dissecting such poorly understood links of metabolism to protein secretion.

In a second demonstration of the power of the SP^SecV^-mRFP reporter and the unusually complex metabolic regulation of protein synthesis and secretion in *S. lividans*, we observed that several growth regimes directly influence exported protein synthesis, partitioning between cytoplasmic and secreted states and secretion kinetics (Figure 3D). Secretome dynamics have become apparent by proteomics studies (Hamed et al., 2017; Tsolis et al., unpublished) but focusing on a single protein here and showing such a range of regulatory events will allow us to directly dissect the molecular basis of the mechanisms. Of interest is the comparison between MM medium in which SP^SecV^-mRFP is synthesized at high-levels but does not get secreted, and NB medium in which high-level synthesis of SP^SecV^-mRFP occurs and it gets gradually secreted at high levels. Fluorescence measurements in both cases demonstrated that a significant population of cytoplasmic SP^SecV^-mRFP becomes folded. This suggests that the secretion capacity has been overcome and would present a bottleneck. The secretion-incompetence of the highly produced SP^SecV^-mRFP in MM (Figure 3E), suggests that perhaps specific factors are required for its secretion and in this growth regime such factors are absent. The eventual loss of cytoplasmic fluorescence in minimal medium is suggestive of additional proteolytic regulatory mechanisms.

Sec-routed eGFP was poorly secreted, this is likely attributable due to its fast folding (Albiniak et al., 2013). The native secretome of TK24 was reduced in complexity and amounts by SP^SecV^-eGFP synthesis/secretion compared to that of SP^SecV^-mRFP. Since most of SP^SecV^-eGFP was converted to folded intracytoplasmic eGFP (Figures 6A,B), either folded eGFP exerts a blockage of some secretory machines, or SP^SecV^-eGFP does engage with translocases but being incompetent for secretion is rapidly ejected. This brief encounter may be sufficient to cause translocase jamming.

*E. coli* remains the premier host for microbial heterologous protein production. However, efficient alternative expression systems are still needed, especially when the protein of interest is required in a soluble form, in the absence of lipopolysaccharide and with low downstream-processing costs. *S. lividans* may be an invaluable non-human pathogen host for recombinant protein secretion using Sec- or Tat-routing.

Our study provides new insight into the complex regulation of protein secretion in Gram-positive bacteria. Combination of fluorescent reporters with transcriptomics, secretomics and metabolomics is anticipated to allow mechanistic understanding of this process.

## Materials and Methods

### Strains and Media Used in the Study

*Streptomyces lividans* TK24 was used as a wild type (Hamed et al., 2017; Koepff et al., 2017). *E. coli* TG1 (Sambrook et al., 1989) served as a host for cloning purposes while *E. coli* S17-1 (Simon et al., 1983) was used for conjugation of DNA from *E. coli* to *Streptomyces*. *E. coli* cultures were grown at 37°C (300 rpm) in Luria Bertani medium, supplemented with ampicillin (50 μg/ml) if applicable. *Streptomyces* cultures are grown at 27–30°C. Protoplast formation and subsequent transformation of *S. lividans* TK24 as well as *E. coli*-*Streptomyces* conjugations were carried out as described (Ausubel et al., 1994; Kieser et al., 2000).

Media used in this study were as described (Hamed et al., 2017):Phage medium (Korn et al., 1978) (per liter: 10 g glucose, 5 g tryptone, 5 g yeast extract, 5 g Lab Lemco powder, 0.74 g CaCl_2_.2H_2_O, 0.5 g MgSO_4_.7H_2_O, pH: 7.2), Minimal Medium (MM) [per liter: 10 g glucose, 3 g (NH_4_)_2_SO_4_, 2.6 g K_2_HPO_4_, 1.8 g NaH_2_PO_4_, 0.6 g MgSO_4_.7H_2_O, 25 mL minor elements solution (per liter: 40 mg ZnSO_4_.7H_2_O, 40 mg FeSO_4_.7H_2_O, 40 mg CaCl_2_, 40 mg MnCl_2_.4H_2_O)], Minimal Medium with 5 g Bacto casamino acids/L (MM_C5_), Bennet medium (Ben) (per liter:10 g Glucose, 2 g tryptone, 1 g yeast extract, 1 g beef extract), Tryptic soy broth (TSB) [per liter: 30 g containing 17 g casein peptone (pancreatic), 5 g NaCl, 3 g soya peptone (papain digest), 2.5 g K_2_HPO_4_, 2.5 g glucose], Nutrient Broth (NB) without NaCl [per liter: 8 g Nutrient Broth pH 6.9, i.e., per L: 5 g peptic digest of animal tissue, 3 g beef extract)], and CM medium (Nowruzi et al., 2008) [per liter: 2.5 g (NH_4_)_2_SO_4_, 10.6 g K_2_HPO_4_, 5.3 g NaH_2_PO_4_, 1 g MgSO_4_.7H_2_O, 1 mL minor elements solution (per liter: 40 g Na_2_EDTA. 2H_2_O, 2 g ZnSO_4_.7H_2_O, 7 g FeSO_4_.7H_2_O, 11 g CaCl_2_, 2 g MnCl_2_.4H_2_O, 0.4 g CuSO_4_.5H_2_O, 0.4 g CoCl_2_.6H_2_O)]. For solid medium, MRYE (Anné et al., 1990) was used supplemented with the appropriate antibiotics, when necessary.

### Standard DNA Manipulations

For all DNA manipulations, standard techniques were followed (Sambrook et al., 1989; Kieser et al., 2000). Oligonucleotides and plasmids are listed in Supplementary Tables S1, S2, respectively. Restriction endonucleases and DNA-modifying enzymes were from Invitrogen and Promega. Standard PCR reactions were carried out using TaKaRa ExTaq (TaKaRa). Generally, PCR-amplified fragments were cloned in pGEM-T Easy and then transferred to the vector of choice by restriction cloning. Oligonucleotides were obtained from Eurogentec S.A., Seraing, Belgium. DNA sequence analysis was carried out according to the dideoxy chain termination method with the Thermo Sequenase Primer Cycle Sequencing Kit with 7-deaza-dGTP (GE Healthcare) on an ALFexpress apparatus (GE Healthcare). As fluorescent-labeled primers, the Cy5-labeled primers M13F and M13R were used. Plasmid DNA was isolated using the WizardPlus SV Miniprep/Midiprep DNA purification System (Promega Inc.). DNA was purified from 1 % agarose gels using the Wizard SV Gel and PCR Clean-Up system (Promega Inc.). As a molecular weight standard for agarose gel electrophoresis the Smart ladder (Eurogentec) was used.

### Vector Constructs

The production of mRFP in *E. coli* was achieved by cloning the mRFP- encoding gene in vector pRSETB with an *N*- terminal hexahistidinyl tag using *a Bam*HI/*Eco*RI restriction fragment to produce pIMBB643 which was over-expressed in BL21 strains harboring the T7 polymerase gene.

To secrete eGFP and mRFP in *S. lividans* via the Sec pathway, the eGFP- *and* mRFP-encoding genes were fused in frame to the *vsi* signal sequence (Van Mellaert et al., 1998a). The eGFP was amplified by PCR from plasmid pIJ8668 (Sun et al., 1999) with the oligonucleotides GFP_For_-GFP_Rev_ and the *mRFP* was amplified by PCR from plasmid pIMBB643 with the oligonucleotides RFP_For_-RFP_Rev_. After cloning the obtained PCR fragments in pGEM-T Easy, the DNA sequences were verified and the resulting pGEMT-eGFP and pGEMT-mRFP plasmids, respectively. pGEMT-eGFP plasmid was digested with *Pst*I, treated with T4 polymerase to remove the 3′-protruding ends and *Eco*RI, while pGEMT-mRFP was digested with *Eco*R47III and *Eco*RI. The resulting DNA fragments were ligated into the pBSDK0.6Sma vector, containing the *vsi* regulatory sequences (Lammertyn et al., 1997), after digestion of this plasmid with *Dra*II, Klenow polymerase treatment and digestion with *Eco*RI. In the resulting plasmids pBSDKmRFP and pBSDKeGFP, the *mRFP* or *eGFP* gene, respectively, was fused to the *vsi* signal sequence preceded by the *vsi* promoter. Both plasmids were digested with *Xba*I/*Hin*dIII and the generated DNA fragments were ligated into the *Xba*I/*Hin*dIII-digested pIJ486 vector, ultimately resulting in the *S. lividans* pIJ486-*sp^SecV^-mRFP* and pIJ486-*sp^SecV^-eGFP* plasmids.

For cloning eGFP and mRFP behind the Tat-dependent *xlnC* signal sequence, the mRFP- and eGFP-encoding genes were amplified by PCR using the previously mentioned templates with the oligonucleotides RFP_For_-RFP_Rev_ and GFP_For_-GFP_Rev_, respectively. After cloning the obtained PCR fragments in pGEM-T Easy as described above, both genes were placed under control of the *vsi* promoter and fused to the *xlnC* signal sequence by cloning the obtained restriction fragment in *Nsi*I/T4 polymerase/*EcoRI*-treated pBSVX (Schaerlaekens et al., 2004b). This resulted in plasmids pBSVXmRFP and pBSVXeGFP, respectively. In the final step, both plasmids were digested with *Xba*I/*Eco*RI and the generated DNA fragments were ligated into the *Xba*I/*Eco*RI-digested pIJ486 vector, ultimately resulting in the *S. lividans* pIJ486-*sp^Tat^-mRFP* and pIJ486-*sp^Tat^*-*eGFP* plasmids.

### Integration of the Gene Encoding sp^SecV^-mRFP in the Chromosome of TK24

The fragment containing the *sp^SecV^-mRFP* gene, i.e., the *S. venezuelae* subtilisin inhibitor signal sequence *sp^SecV^* fused to *mRFP* behind the strong *vsi* promotor (*P_vsi_*), was excised from the plasmid pIJ486-sp^SecV^-mRFP using *Xba*I and *Hind*III and ligated into the respective sites of pTOS (Herrmann et al., 2012) yielding pTOS+mRFP that contained the sp^SecV^-mRFP-encoding gene and *attP* of VWB phage flanked by two *rox*-sites. This plasmid was introduced into the genome of *S. lividans* TK24 by intergeneric conjugation with *E. coli* ET1326::pUZ8002 (Kieser et al., 2000). Genomic DNAs of four randomly chosen exconjugants were isolated and verified by PCR for proper integration of the pTOS+mRFP plasmid. Then, the pUWLDre plasmid containing the gene of the *Dre* recombinase, was introduced into the respective mutant strain and the pTOS+mRFP-backbone was excised as described elsewhere (Herrmann et al., 2012).

### Growth Conditions

*Streptomyces lividans* TK24 and its derivatives were precultured in 50 ml Phage medium (Korn et al., 1978) supplemented with thiostrepton (10 μg/ml) if necessary, and grown at 28°C with continuous shaking at 240 rpm for 48 h. After growth, the optical density of preculture was measured at 600 nm (OD_600_) and the mycelia were harvested by centrifugation (3800 × *g*; 15 min; SIGMA 3–16 K centrifuge) and washed twice with sterilized water. After homogenizing the mycelium in 50 mL of sterilized water, the strains were inoculated into 250-mL Erlenmeyer flasks containing 100 mL of media that were described above. In order to have the same amount of mycelia per each inoculum this equation was used: the volume of inoculum = (Final volume of culture × 0.25)/ OD_600_ (Gomez-Escribano and Bibb, 2012). The flasks were shaken at 240 rpm and 28°C, and pH was controlled using 100 mM MES buffer (pH 6.9).

### Dry Cell Mass Determination

To quantify the dry cell weight (DCW), 10 mL of culture was centrifuged at 3800 × *g* for 15 min (SIGMA 3–16 KL refrigerated centrifuge). The bacterial pellets were harvested, resuspended in sterilized water and filtered under vacuum using a 0.2 μm pore size filter (predried and preweighted; PORAFIL^®^ MV; Macherey-Nagel). The filter was once more dried (overnight 12–24 h in an oven at 60°C) and weighted for DCW determination.

### Cell Growth in a Micro-Bioreactor and Online Fluorescence Monitoring

Use of a micro-bioreactor has been described (Koepff et al., 2017). Briefly, 24-well plates (Greiner Bio One) were filled with 1 ml of NB or CM medium, seeded with 50 μl of an *S. lividans* preculture and incubated at 29°C while shaking (4 mm, orbital). During growth, fluorescence was measured every 15 min.

### Fluorescence Assays and Quantification of Fluorescent Proteins

Fluorescence measurements were carried out in an Infinite^®^ M200 microplate reader (Tecan) (eGFP: excitation 485 nm/emission 510 nm; mRFP: excitation at 550 nm/emission at 580 nm). To compare strain performance, mRFP and eGFP fluorescence intensities, obtained at the transition to stationary phases were identified and evaluated. Error propagation was applied to calculate DCW-specific mRFP and eGFP production.

The quantification of eGFP and mRFP was carried out by cloning *mRFP* and *eGFP* as His-tagged versions in pIMBB316 and pIMBB643, respectively. Plasmids were transformed in *E. coli* strain BL21 followed by purification of His-eGFP and His-mRFP by metal affinity chromatography (not shown). Calibration curves of both fluorescence measurements and protein mass were generated using titrations of protein amounts (Supplementary Figures S1A,B). The amounts of eGFP and mRFP detected via fluorescence assays were compared with the amounts quantified via western blotting (see western immuno-blotting analysis below).

### SDS–PAGE and Western Blot Analysis

Extracellular protein fractions of cultures of *S. lividans* and its derivatives were obtained by centrifugation (10 min, 4200 × *g*, 4°C). Precipitation of the proteins in the supernatant was, where applicable, carried out with trichloroacetic acid (TCA) (final concentration of 20% w/v; 4°C). Proteins were separated by SDS–PAGE and as a standard the Precision Plus Protein^TM^ Standard (All Blue) from Bio-Rad was used. Proteins were visualized by Coomassie Brilliant Blue (CBB) or by Western blotting and immuno-detection with specific antibodies in combination with a suitable secondary alkaline phosphatase-conjugated antibody (Sigma). eGFP and mRFP antibodies were obtained from Immunosource or generated against lab-purified proteins at Davids Biotechnologie, Germany. Quantification of expression signals on the blot was carried using ImageJ software.

### Miscellaneous

Chemicals were from Sigma. Bacto Soytone from DIFCO Laboratories. DNA enzymes were from New England Biolabs and oligonucleotides from Eurogentec. Images were scanned in an ImageQuant^TM^ 300 or a LAS4000 system and analyzed using the ImageQuant TL software to compare the intensity of the protein bands to those of the BSA calibration bands or other control proteins. SDS–PAGE and western blotting is described in the Supplemental Materials.

## Author Contributions

MH and KV performed the experiments, cell growth, and analyzed the data. KV, BB, LM, RN, JaK, and AL constructed the clones. JoK and MO performed the cell fitness analyses. JA analyzed the data. AE and SK conceived, managed and supervised the project, and wrote the paper with contribution from MH. All authors read and approved the manuscript.

## Conflict of Interest Statement

The authors declare that the research was conducted in the absence of any commercial or financial relationships that could be construed as a potential conflict of interest.
