# Supplementary material for: Monitoring Protein Secretion in Streptomyces Using Fluorescent Proteins
Source: Front Microbiol. 2018 Dec 7;9:3019. doi: 10.3389/fmicb.2018.03019 (PMC6292873; doi:10.3389/fmicb.2018.03019)
Supplement: Supplementary file 1 [file Data_Sheet_1.docx]

Supplemental Materials:

Monitoring protein secretion in *Streptomyces* using fluorescent proteins

Hamed, Mohamed Belal^1,6^; Vranken, Kristof^1^*, Bilyk, Bohdan^2^, Koepff, Joachim^3^**, [Nováková, Renáta](https://www.sav.sk/index.php?lang=en&doc=user-org-user&user_no=1322)^4^, Van Mellaert, Lieve^1^, Oldiges, Marco^3^, Luzhetskyy, Andriy^5^, Kormanec, Jan^4^, Anné, Jozef^1^, Karamanou, Spyridoula^1^ and Economou, Anastassios^1,7^

^1^ KU Leuven, Rega Institute, Dpt of Microbiology and Immunology, Herestraat 49, B-3000 Leuven, Belgium

^2^ PharmBioTec GmbH, Science Park 1, 66123, Saarbrücken, Germany

^3^ Forschungszentrum Jülich GmbH, Institute of Bio- and Geosciences, IBG-1: Biotechnology, Leo-Brandt-Straße, 52428, Jülich, Germany.

^4^ Institute of Molecular Biology, Slovak Academy of Sciences, Dubravska cesta 21, 84551 Bratislava, Slovakia

^5^ Helmholtz-Zentrum für Infektionsforschung GmbH, Inhoffenstraße 7, 38124 Braunschweig, Germany

^6^ Molecular Biology Dpt, National Research Centre, Dokii, Cairo, Egypt

Present address:

* Citrique Belge, Pastorijstraat 249, B-3300 Tienen, Belgium

** Sartorius Stedim Biotech GmbH, August-Spindler-Strasse 11, 37079 Göttingen

^7^ For correspondence:

e-mail: [tassos.economou@kuleuven.be](mailto:tassos.economou@kuleuven.be)

Running title: Streptomyces fluorescent reporter secretion

Supplemental Materials contents:

Supplementary materials and methods

Supplementary figures

**Figure S1** Calibration curves for mRFP and eGFP, restriction map of the *Streptomyces* integrative plasmid pAPHII14-mCherry and comparison of the percentage of intracellular folded and released mCherry.

**Figure S2** Integration of the gene encoding for *sp^SecV^-mRFP* into the chromosome of *S. lividans* TK24.

**Figure S3** Schematic representation of the construction of the *lsi*(*SLIV_34120)-mRFP* fusion cassette.

**Figure S4** Southern blot verification of the chromosomal integration of *sp^SecL^-mRFP*.

**Figure S5** Integration of the gene encoding for *sp^SecL^-mRFP* into the chromosome of *S. lividans* TK24.

**Figure S6** Optimizing mRFP secretion.

Supplementary tables

**Table S1** Oligonucleotides used in this study.

**Table S2** Plasmids used in this study.

**References**

Supplementary materials and methods

*Quantification of folded and unfolded intracellular fluorescent proteins*

The quantification of eGFP and mRFP were carried out using the fluorescence intensities of specific protein masses (see materials and methods) and considered as folded amounts. The total folded and unfolded proteins were estimated by quantifying the expression signals of western blot using calibrations curves of signals and protein masses of purified proteins and analyzed by ImageJ software. The unfolded proteins amount were calculated by subtracting the total by the folded amounts.

*Integration of the sp^SecL^-mRFP gene into the chromosome of S. lividans TK24 via homologous recombination*

Based on the analysis of the secretome of *S. lividans* TK24 ([Hamed et al., 2017](#_ENREF_3); [Tsolis et al., Submitted](#_ENREF_12)) the *SLIV_34120* gene (*S. lividans subtilisin inhibitor* or *lsi,* also known as *sti1*) product is secreted at high levels. This gene is homologous to the *vsi* gene (*S. venezuelae* subtilisin *inhibitor*) we have extensively used before to over-express and secrete heterologous polypeptides ([Lammertyn et al., 1997](#_ENREF_8)). The signal peptide encoded by *lsi* (SP^SecL^) was fused upstream of *mRFP* to generate *sp^SecL^-mRFP* (Fig. S3). For the integration of the gene fusion into the chromosome of *S. lividans* TK24 we used the pAMR4-based deletion system for streptomycetes that relies on the positive selection of double crossover events using the blue pigment-producing gene *bpsA*1).

First, we PCR-amplified a 2-kb DNA fragment upstream of *lsi* with an inserted *Nhe*I restriction site downstream of the signal peptide processing site using the primers 34120Spe and 34120Nhe (Table S1). This DNA fragment was digested using *Spe*I and *Nhe*I and cloned into pAMR4 (1), digested with the same restriction enzymes, resulting in pAMR4-sec1A. Subsequently the *mRFP* reporter gene was PCR-amplified with the primers mRFPNhe and mRFPNdeI (Table S1) using the plasmid pIJ486-sp^SecV^-mRFP as a template. The DNA fragment was digested with *Nhe*I and *Nde*I and cloned into pAMR4-sec1A digested with the same enzymes, resulting in pAMR4-sec1AR. Finally, a 2-kb DNA fragment downstream of the *lsi(SLIV_34120)* gene was PCR amplified using the primers 34120Afl and 34120Hind (Table S1), the DNA fragment was digested using *Afl*II and *Hin*dIII and cloned into pAMR4-sec1AR cut with the same enzymes, resulting in pAMR4-secA2AR (Fig. S3). The correct fusion and cloning in the final plasmid was confirmed by nucleotide sequencing. The plasmid was transformed into the non-methylating *E. coli* ET12567/pUZ8002 strain and then introduced into *S. lividans* TK24 by conjugation with selection for apramycin resistance. Three white (i.e. *bpsA*-negative) apramycin-resistant and kanamycin-sensitive colonies (indicating a double cross-over event) were selected, and the correct replacement of the *lsi (SLIV_34120)* gene was confirmed by Southern-blot hybridization (Fig. S4). The recombinant strain *S. lividans*, D34120-AR selected colonies, with *lsi* (*SLIV_34120)* gene replaced by the *mRFP* fused to its signal peptide, were phenotypically similar in growth and sporulation to *S. lividans* TK24.

*Construction of the integration plasmid vector pAPHII14-mCherry containing the mCherry gene, encoding an improved variant of mRFP, under the control of strong ermEp* promoter.*

The *ermEp** promoter-bearing DNA fragment was PCR amplified using primers ErmBam_For_ and ErmKpn_Rev_ (Table S1) and pErmEp3 plasmid ([Novakova et al., 2011](#_ENREF_9)) as a template. The 300-bp DNA fragment was digested with *Kpn*I and *Bam*HI.and cloned in pMU1s* plasmid vector ([Craney et al., 2007](#_ENREF_2)) digested with the same restriction enzymes, resulting in pMU1s-ermEp4. Subsequently, the *mCherry* gene, encoding an improved variant of mRFP, was amplified from the pmCherry template plasmid (Clontech, Mountain view, CA, USA) with the primers mCherryNde and mCherryNot (Table S1), the 720-bp DNA fragment was digested with *Nde*I and *Not*I and cloned in pMU1s-ermEp4 digested with the same restriction enzymes, resulting in pErmEp-mCherry. Finally, 1500-bp EcoRI (filled with Klenow enzyme) – *Bam*HI DNA fragment from pErmEp-mCherry was cloned in the *Streptomyces* integrative plasmid pAPHII14 ([Novakova et al., 2011](#_ENREF_9)) digested with *Xho*I (filled with Klenow enzyme) – *Bam*HI, resulting in pAPHII14-mCherry. All DNA fragments and fusions were verified by nucleotide sequencing. The plasmid was introduced into *S. lividans* TK24 chromosome by standard conjugation procedure ([Kieser et al., 2000](#_ENREF_5)). Kanamycin-resistant clones were selected and used for measurement of intracellular and extracellular mCherry fluorescence activity.

Supplementary figures:


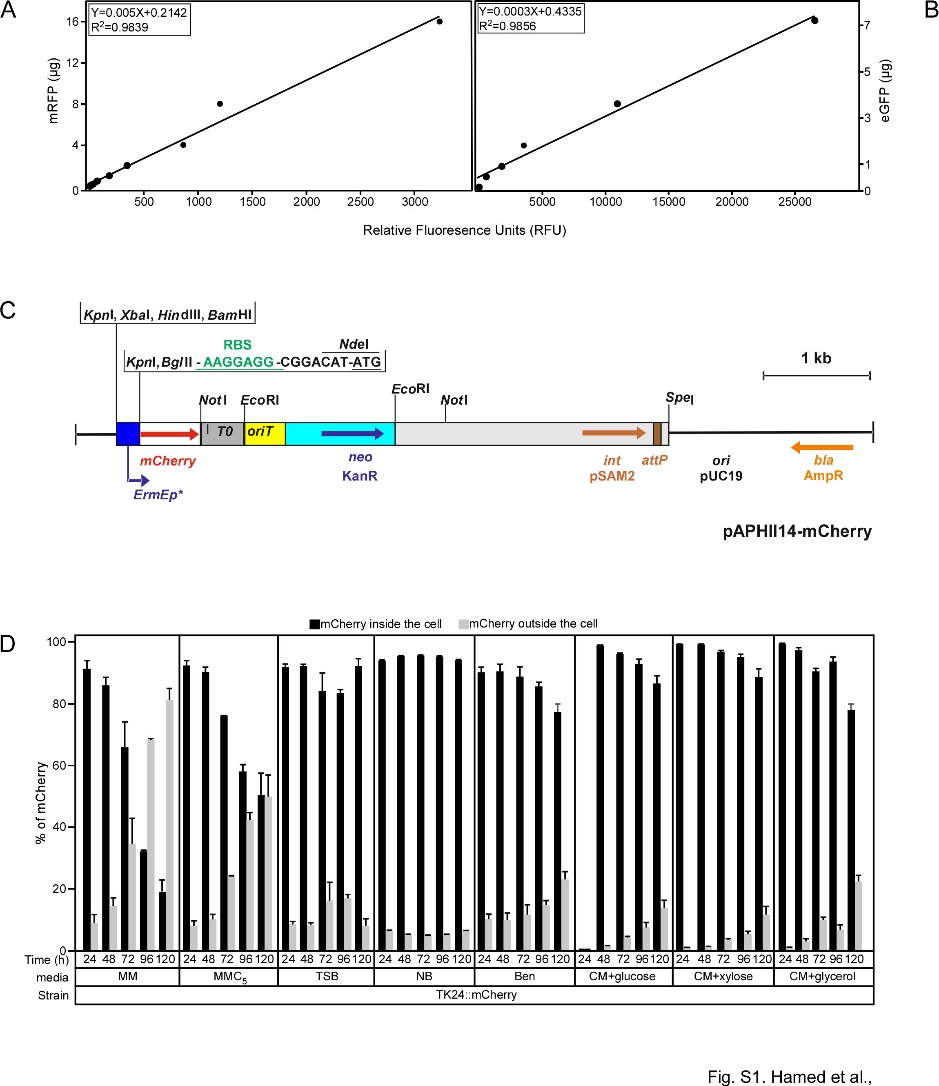


**Figure S1:** Calibration curves for mRFP and eGFP, restriction map of the *Streptomyces* integrative plasmid pAPHII14-mCherry and comparison of the percentage of intracellular folded and released mCherry.

**(A)** and **(B)** Calibration curves employed for the correlation of RFU to specific amounts of the fluorescence proteins **(A)** mRFP and **(B)** eGFP. Fluorescence intensity was measured with excitation wavelengths of 550 nm and 485 nm and emission at 580 nm and 510 nm for mRFP and eGFP, respectively.

**(C)** Restriction map of the *Streptomyces* integrative plasmid pAPHII14-mCherry.

pAPHII14 plasmid contains the *mCherry* gene, encoding an improved variant of mRFP, under the control of strong *ermEp** promoter and strong RBS site (AAGGAGG), λ*T0* terminator, *oriT* origin of transfer from the broad-host-range plasmid RK2, the kanamycin-resistance gene *neo* (KanR) from Tn*5,* integrase-encoding gene *int* with the *attP* site from the plasmid pSAM2, the *E. coli* ColE1 replication origin and the ampicillin resistance gene *bla* (AmpR) from pBluescript II SK+. The relevant restriction sites are indicated.

**(D)** Comparison of the percentage of folded mCherry both intracellular and secreted.

The percentage of intracellular folded signal peptide-less mCherry expressed by *S. lividans* TK24 (black) compared with the percentage of signal peptide-less mCherry found in the spent growth medium and which derives from cell lysis (gray). Relative fluorescence units (RFU) for mCherry, were determined by measuring the emission at 600 nm after excitation at 570 nm.

**
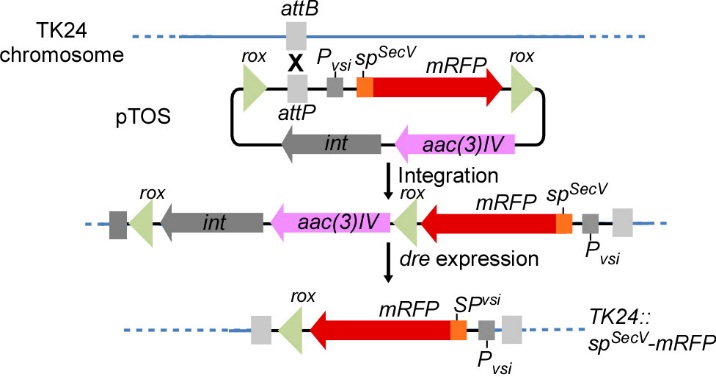
**

**Figure S2.** Integration of the gene encoding for *sp^SecV^-mRFP* into the chromosome of *S. lividans* TK24.

The phage VWB integrase catalyzes integration of the pTOS+*sp^SecV^-mRFP* into the VWB attachment site of *S. lividans* TK24 (i). The plasmid is integrated into the genome and selection for the plasmid is provided by *aac(3)IV* encoding the apramycin resistance marker (ii). After expression of the *dre* recombinase, the region flanked by two *rox* sites is deleted thus leaving behind only the *sp^SecV^-mRFP*-encoding gene and one *rox* site in the genome (iii).


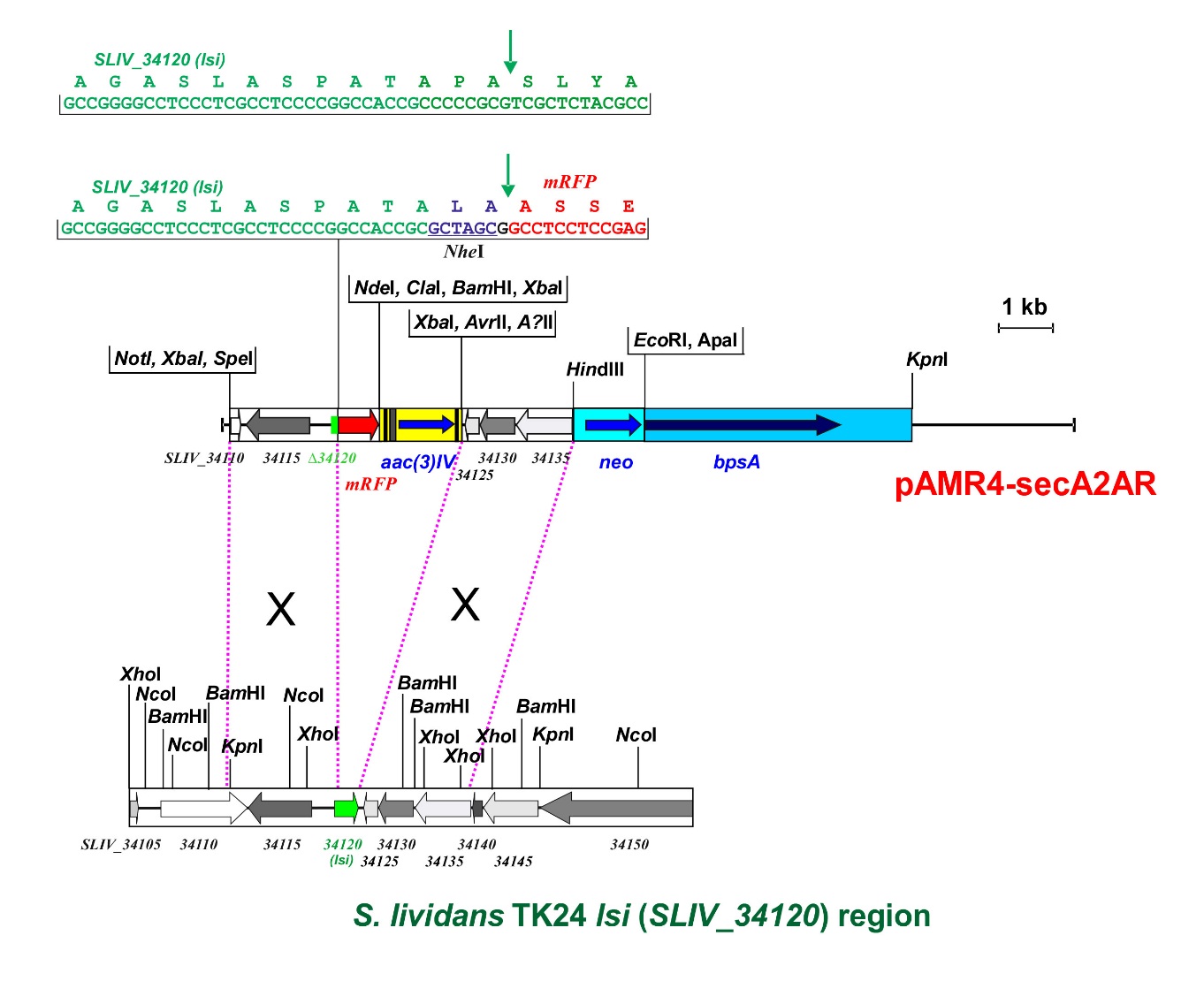


Figure S3 Scheme of the construction of the *sp^SecL^-mRFP* fusion cassette.

The PCR product of 2-kb DNA fragments from *S. lividans* TK24 (the upstream and downstream region of *the lsi* gene (*SLIV_34120*) were cloned in pAMR4 (1), resulting pAMR4-secA2AR. The nucleotide and amino acid sequence of the partial signal sequence for *lsi* and the fusion to *mRFP* through an inserted *Nhe*I site are indicated. The arrow indicates the predicted signal peptidase cleavage site (2). See Supplementary Materials and methods for details. The promoter-less *bpsA* gene, the kanamycin resistance gene from Tn*5* (*neo*), and the apramycin resistance gene *aac3(IV)* with *oriT* and (flippase recognition target) FRT regions of plasmid pAMR4 are also indicated.


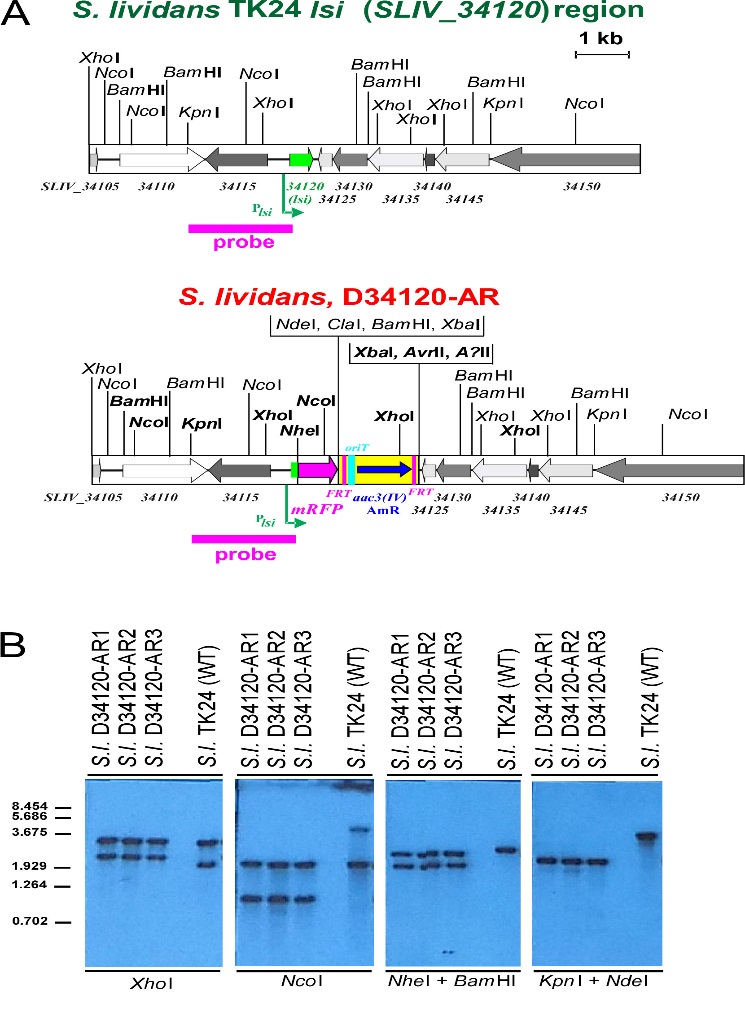


Figure S4: Southern blot verification of *sp^SecL^-mRFP* chromosomal integration

(A) Physical maps of chromosomal DNA containing the wild-type *S. lividans* TK24 *lsi*(*SLIV_34120)* region, and the *S. lividans*, D34120-AR mutant strain with replaced *lsi*(*SLIV_34120)* gene by a fusion containing its signal sequence fused to *mRFP.* Thick arrows denote the direction and size of genes; green arrow indicates the *lsi*(*SLIV_34120)* gene; red arrow represents the *mRFP* gene. Gene labelling is based on the genomic sequence of *S. lividans* TK24 (GenBank Acc. No. CP009124) (3). The pink bar below the maps represents the probe used for Southern hybridization analysis. Relevant restriction sites are indicated. (B) Southern hybridization analysis of chromosomal DNA from the indicated strains verifying the correct integration. 1 μg of DNA from the corresponding strain was digested with the restriction enzymes indicated, separated by electrophoresis in 0.8% (w/v) agarose gel and transferred on Hybond N (Amersham) as described in (4). Hybridization followed the standard DIG protocol (Roche, Mannheim, Germany) using the DIG-labelled probe for verification of correct integration. Lambda DNA-*Bst*EII digest was used as the size standard.

**
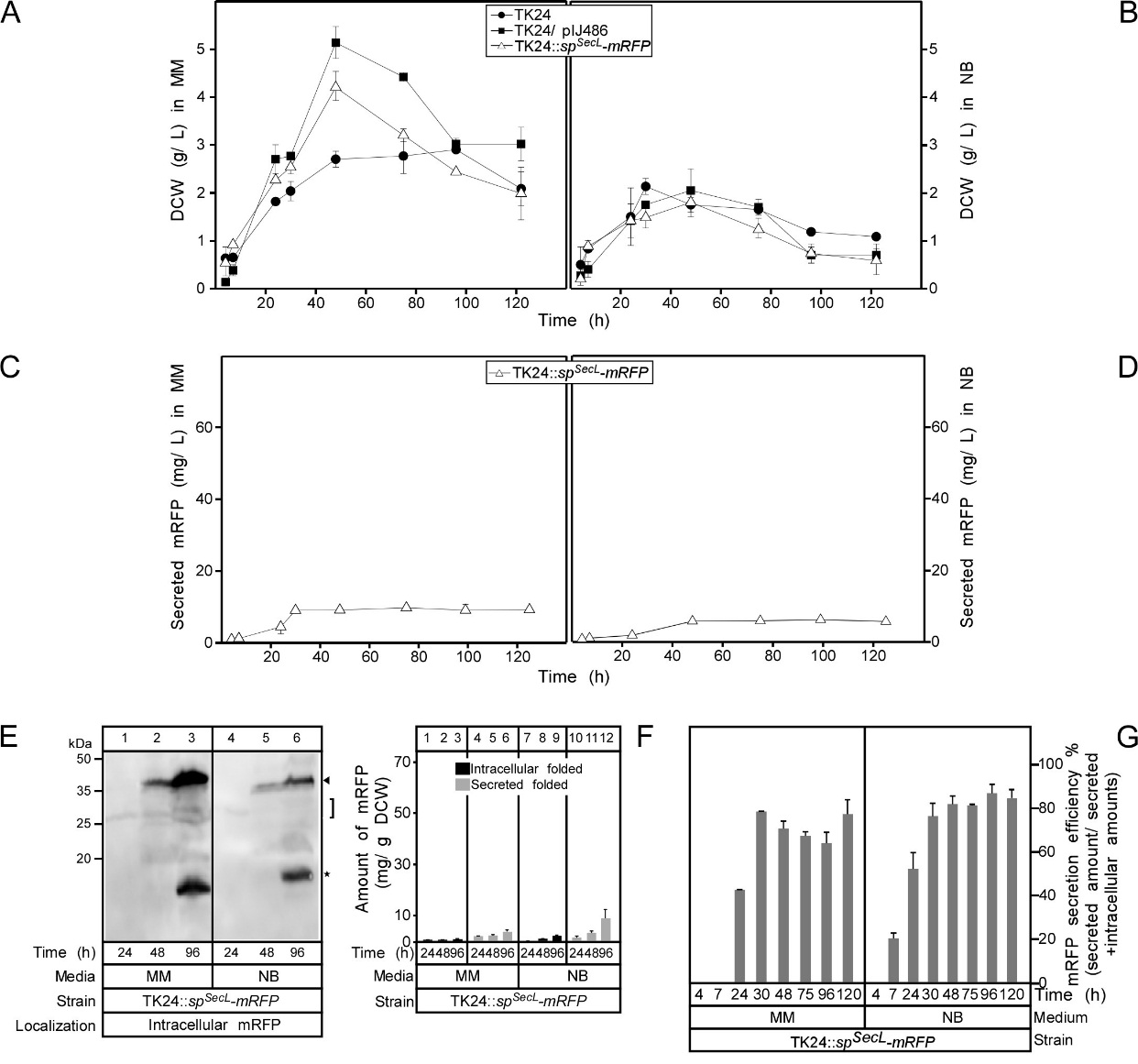
**

**Figure S5:** Integration of the gene encoding for *sp^SecL^-mRFP* into the chromosome of *S. lividans* TK24.

**(A)** and **(B)** Comparison of cell growth of *S. lividans* TK24 as a wild type and TK24::*sp^SecL^-mRFP* or TK24/ pIJ486 plasmid in minimal medium (MM) and nutrient broth (NB) expressed as values of dry cell weight (DCW) (g/L). *n*=3, values represent mean ± SD.

**(C)** and **(D)** the amounts of mRFP secreted (in mg/L) from TK24::*sp^SecL^-mRFP* in minimal medium (MM) and nutrient broth (NB) for the indicated time related to its growth curves in the same media. *n*=3, values represent mean ± SD.

**(E)** Western blot analysis immuno-probed for intracellular mRFP with α-mRFP antibodies in TK24 grown in minimal medium (MM) and nutrient broth (NB). Total cell lysates loaded are equivalent to 0.6 mg of dry cell mass of TK24::*sp^SecL^-mRFP* grown for the indicated times in the indicated media, washed twice and loaded on 12% SDS-PAGE and visualized using α-mRFP antibodies. Intact mRFP is indicated (filled arrow); stars indicate degradation of mRFP.

**(F)** Comparison of the yield of secreted and intracytoplasmic folded mRFP (in mg) correlated to a gram of dry cell mass from TK24::*sp^SecL^-mRFP* grown in minimal medium (MM) and nutrient broth (NB) for the indicated time related to its growth curves in the same media. *n*=3, values represent mean ± SD.

**(G)** Secretion efficiency, expressed as % of the total detectable secreted and folded mRFP as a fraction of the total synthesized SP^SecL^-mRFP (folded secreted+folded cytoplasmic+non-folded cytoplasmic), of TK24::*sp^SecL^-mRFP* in minimal medium (MM) and nutrient broth (NB) for the indicated time related to its growth curves in the same media. *n*=3, values represent mean ± SD.


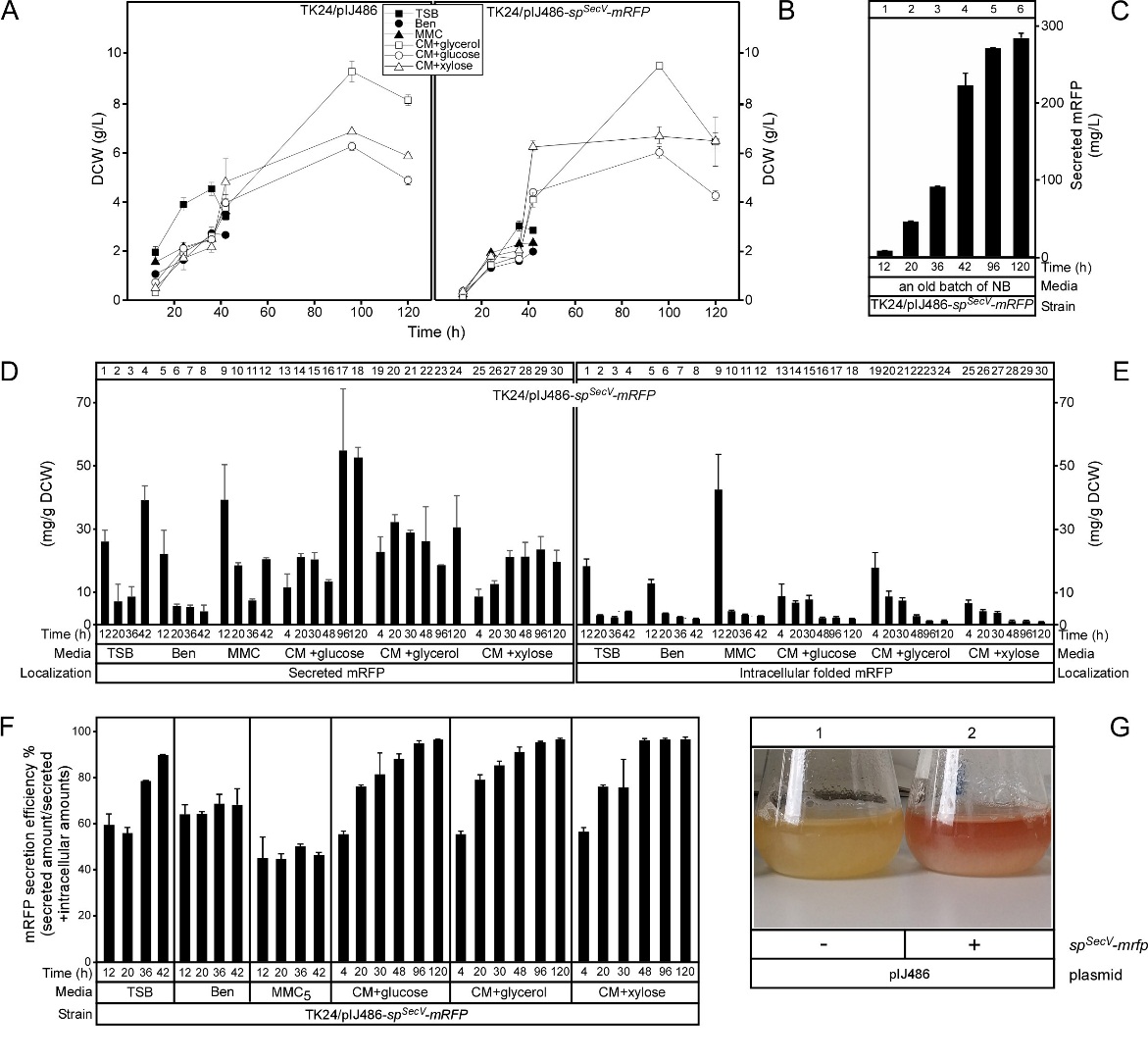


**Figure S6**: Optimizing mRFP secretion

**(A)** and **(B)** Comparison of cell growth of *S. lividans* TK24/pIJ486 or TK24/pIJ486-*sp^SecV^-mRFP* in different media. TSB=tryptic soy broth; Ben= Bennet medium; MMC= minimal medium supplemented with casamino acids (5 g/L) NB=nutrient broth; CM= complete medium supplemented with different carbon sources (glucose, glycerol and xylose) (16 g/L). The nitrogen source for this medium had previously been optimized (5). **(C)** The amount of folded secreted mRFP determined in (mg/L) produced by TK24/pIJ486-*sp^SecV^-mRFP* (High production level) in a different batch of nutrient broth medium (NB) than that used in this study. *n*=3, values represent mean ± SD.

**(D) and (E)** The amount of folded secreted **(D)** and intracytoplasmic **(E)** mRFP in (mg) correlated to a gram of dry cell weight (DCW) produced by TK24/pIJ486-*sp^SecV^-mRFP* (High production level) in the indicated media and time, related to its growth curve in the same media. *n*=3, values represent the mean ± SD.

**(F)** Secretion efficiency, expressed as % of the total detectable secreted and folded mRFP as a fraction of the total synthesized SP^SecV^-mRFP (folded secreted+folded cytoplasmic), of TK24/pIJ486-*sp^SecV^-mRFP* (High production level) in indicated media for the indicated time related to its growth curves in the same media. *n*=3, values represent mean ± SD.

**(G)** Difference in culture color between an *S. lividans* TK24/pIJ486 (left) and an *S. lividans* TK24/pIJ486-*sp^SecV^-mRFP* (right) colony when inoculated and grown in NB medium for 48h.

Supplemental Tables

**Table S1: Oligonucleotides used in this study**

| **Name** | **Sequence (5’- 3’ direction)** | **Restriction sites** |
| --- | --- | --- |
| RFP_For_ | TAAGCGCTTCCTCCGAGGACGTCATC | *Eco*R47III |
| RFP_Rev_ | ATGAATTC**CTA**GGCGCCGGTGGAGTGG | *Eco*RI |
| GFP_For_ | ATCTGCAGTGAGCAAGGGCGAGGAGC | *Pst*I |
| GFP_Rev_ | ATGAATTC**CTATTA**CTTGTACAGCTCGTCC | *Eco*RI |
| 34120Spe | CCCCCACTAGTGCTGTGTCTGGACTCGCGGGCGG | *Spe*I |
| 34120Nhe | CCCCCGCTAGCGCGGTGGCCGGGGAGGCGAGGG | *Nhe*I |
| 34120Afl | CCCCCCTTAAGCTCCCGGTTCAGGGGCACGGTCGG | *Afl*II |
| 34120Hind | CCCCCAAGCTTCATGGAGTACGACCGCGAAGGACC | *Hin*dIII |
| mRFPNheI | CCCCCGCTAGCGGCCTCCTCCGAGGACGTCATCAAG | *Nhe*I |
| mRFPNdeI | CCCCCCATATGTCCTAGGCGCCGGTGGAGTGGCGG | *Nde*I |
| ErmBam_For_ | CCCCGGATCCAGCCCGACCCGAGCACG | *Bam*HI |
| ErmKpn_Rev_ | CCCCGGTACCGATCCTACCAACCGGCACG | *Kpn*I |
| mCherryNde | CCCCCATATGGTGAGCAAGGGCGAGGAGG | *Nde*I |
| mCherryNot | CCCCCCGCGGCCGCTACTTGTACAGCTCGTCCATGC | *Not*I |

Restriction endonuclease cleavage sites are underlined, stop codons are indicated in bold.

**Table S2: Plasmids used in this study**

| **Name** | **Relevant properties** | **References** |
| --- | --- | --- |
| *E. coli* plasmids | | |
| pGEM-T Easy | Multiple cloning site, *bla* | Promega |
| pRSETB | Expression vector containing the N-terminal hexahistidinyl tag mRFP gene. | This study |
| pBSDK0.6Sma | pBluescript KS(+) derivative containing the *S. venezuelae* vsi promoter and signal sequence | ([Lammertyn, 2000](#_ENREF_7)) |
| pBSVX | pBluescript KS(+) derivative containing the *S. venezuelae* *vsi* promoter and the signal sequence of *S. lividans* *xlnC* | ([Schaerlaekens et al., 2004](#_ENREF_10)) |
| pBSV/*sp^Tat^-eGFP* | pBSVX derivative containing the *S. venezuelae* *vsi* promoter, followed by the *S. lividans xlnC* signal sequence and the eGFP cDNA. | This study |
| pBSV/*sp^Tat^-mRFP* | pBSVX derivative containing the *S. venezuelae vsi* promoter, followed by the *S. lividans xlnC* signal sequence and the mRFP cDNA. | This study |
| pBSDK/*sp^SecV^­-mRFP* | pBSDK0.6Sma derivative containing the *S. venezuelae vsi* promoter and signal sequence and the mRFP cDNA. | This study |
| pErmEp3 | LITMUS 28 derivative containing ermEp* promoter. | ([Novakova et al., 2011](#_ENREF_9)) |
| pMU1s-ermEp4 | pMU1s derivative containing ermEp* promoter and apramycin resistant gene *aac(3)IV.* | ([Craney et al., 2007](#_ENREF_2)) |
| pmCherry | Plasmid contains mCherry gene. The vector backbone contains a pUC origin of replication for propagation in *E. coli*, and an f1 origin for single-stranded DNA production. A neomycin-resistance cassette (Neo^r^). | This study |
| pEmrEp-mCherry | pMU1s-ermEp4 derivative containing mCherry gene. | This study |
| *E. coli* 🡪 *Streptomyces* conjugational plasmids | | |
| pIJ8668 | pIJ8600 derivative containing the eGFP cDNA. | ([Sun et al., 1999](#_ENREF_11)) |
| pAMR4 | It is a pBluescript II SK+ backbone containing the promoterless *bpsA* gene, the kanamycin resistance gene from Tn5 (*neo*), and the apramycin resistance gene *aac3 (IV)* with *oriT* and *FRT* regions from the plasmid pIJ773. | ([Knirschova et al., 2015](#_ENREF_6)) |
| pTOS | pSOK804 derivative; *attP* flanked by *rox* sites | ([Helmann, 2002](#_ENREF_4)) |
| pTOS/*sp^SecV^-mRFP* | pTOS derivative containing the *S. venezuelae vsi* promoter, followed by the *S. lividans xlnC* signal sequence and the mRFP cDNA. |  |
| pUWLDre | pUWLoriT derivative with the *dre* gene under an *ermE* promoter | ([Helmann, 2002](#_ENREF_4)) |
| pAPHII14-mCherry | An integrative vector of pAPHII14 derivative containing mCherry gene. | ([Novakova et al., 2011](#_ENREF_9)) |
| *Streptomyces* plasmids | | |
| pSVH1 | *Streptomyces spp*. *oriC* | ([Bierman et al., 1992](#_ENREF_1)) |
| pIJ486 | Multiple cloning site, Tsr^R^ | ([Ward et al., 1986](#_ENREF_13)) |
| pIJ486-*sp^SecV^-mRFP* | Derivative of pIJ486 containing *S. venezuelae vsi* promoter and signal sequence fused in frame to the mRFP cDNA. | This study |
| pIJ486-*sp^Tat^-mRFP* | Derivative of pIJ486 containing *S. venezuelae vsi* promoter and *xlnC* signal sequence fused in frame to the mRFP cDNA. | This study |
| pIJ486*-sp^SecV^*-eGFP | Derivative of pIJ486 containing *S. venezuelae vsi* promoter and signal sequence fused in frame to the eGFP cDNA. | This study |
| pIJ486-*sp^Tat^-*eGFP | Derivative of pIJ486 containing *S. venezuelae vsi* promoter and *xlnC* signal sequence fused in frame to the eGFP cDNA. | This study |

**References:**

Bierman, M., Logan, R., O'Brien, K., Seno, E.T., Rao, R.N., and Schoner, B.E. (1992). Plasmid cloning vectors for the conjugal transfer of DNA from *Escherichia coli* to *Streptomyces spp*. *Gene* 116(1)**,** 43-49.

Craney, A., Hohenauer, T., Xu, Y., Navani, N.K., Li, Y., and Nodwell, J. (2007). A synthetic luxCDABE gene cluster optimized for expression in high-GC bacteria. *Nucleic Acids Res* 35(6)**,** e46. doi: 10.1093/nar/gkm086.

Hamed, M.B., Karamanou, S., Olafsdottir, S., Basilio, J.S.M., Simoens, K., Tsolis, K.C., et al. (2017). Large-scale production of a thermostable *Rhodothermus marinus* cellulase by heterologous secretion from *Streptomyces lividans*. *Microb Cell Fact* 16(1)**,** 232. doi: 10.1186/s12934-017-0847-x.

Helmann, J.D. (2002). The extracytoplasmic function (ECF) sigma factors. *Adv Microb Physiol* 46**,** 47-110.

Kieser, T., Bibb, M.J., Buttner, M.J., Chater, K.F., and Hopwood, D.A. (2000). *Practical Streptomyces genetics.* Norwich, UK.: John Innes Foundation.

Knirschova, R., Novakova, R., Mingyar, E., Bekeova, C., Homerova, D., and Kormanec, J. (2015). Utilization of a reporter system based on the blue pigment indigoidine biosynthetic gene bpsA for detection of promoter activity and deletion of genes in Streptomyces. *J Microbiol Methods* 113**,** 1-3. doi: 10.1016/j.mimet.2015.03.017.

Lammertyn, E. (2000). Isolation and characterization of a novel subtilisin inhibitor from *Streptomyces venezuelae*  and evaluation of its regulatory sequences for heterologous protein production by *Streptomyces lividans*. K.U.Leuven, Belgium, PhD thesis. pp 148.

Lammertyn, E., Van Mellaert, L., Schacht, S., Dillen, C., Sablon, E., Van Broekhoven, A., et al. (1997). Evaluation of a novel subtilisin inhibitor gene and mutant derivatives for the expression and secretion of mouse tumor necrosis factor alpha by *Streptomyces lividans*. *Appl Environ Microbiol* 63(5)**,** 1808-1813.

Novakova, R., Rehakova, A., Feckova, L., Kutas, P., Knischova, R., and Kormanec, J. (2011). Genetic manipulation of pathway regulation for overproduction of angucycline-like antibiotic auricin in *Streptomyces aureofaciens* CCM 3239. *Folia Microbiol (Praha)* 56(3)**,** 276-282. doi: 10.1007/s12223-011-0033-1.

Schaerlaekens, K., Van Mellaert, L., Lammertyn, E., Geukens, N., and Anné, J. (2004). The importance of the Tat-dependent protein secretion pathway in *Streptomyces* as revealed by phenotypic changes in tat deletion mutants and genome analysis. *Microbiology* 150(Pt 1)**,** 21-31. doi: 10.1099/mic.0.26684-0.

Sun, J., Kelemen, G.H., Fernandez-Abalos, J.M., and Bibb, M.J. (1999). Green fluorescent protein as a reporter for spatial and temporal gene expression in *Streptomyces coelicolor A3*(2). *Microbiology* 145 ( Pt 9)**,** 2221-2227. doi: 10.1099/00221287-145-9-2221.

Ward, J.M., Janssen, G.R., Kieser, T., Bibb, M.J., Buttner, M.J., and Bibb, M.J. (1986). Construction and characterisation of a series of multi-copy promoter-probe plasmid vectors for *Streptomyces* using the aminoglycoside phosphotransferase gene from Tn5 as indicator. *Mol Gen Genet* 203(3)**,** 468-478.
